# Investigating the causal relationship between thyroid dysfunction diseases and osteoporosis: a two-sample Mendelian randomization analysis

**DOI:** 10.1038/s41598-024-62854-x

**Published:** 2024-06-04

**Authors:** Weihui Qi, Dong Wang, Yihu Hong, Jun Yao, Huang Wang, Li Zhu, Hao Pan

**Affiliations:** 1 Department of Orthopaedics, Hangzhou TCM Hospital Affiliated to Zhejiang Chinese Medical University, Hangzhou, China; 2 Department of Orthopaedics, Hangzhou Ding Qiao Hospital, Hangzhou, China

**Keywords:** Two-sample Mendelian randomization, Osteoporosis, Thyroid dysfunction diseases, Inverse variance weighted, Endocrine system and metabolic diseases, Risk factors, Genetic association study

## Abstract

The prevalence of thyroid dysfunction diseases (TDFDs) and osteoporosis (OP) is high. Previous studies have indicated a potential association between TDFDs and OP, yet the causal direction remains unclear. This study aimed to investigate the potential causal relationship between TDFDs and the risk of developing OP and related fractures. We obtained pooled data from genome-wide association studies (GWASs) conducted on TDFDs and OP in European populations and identified single-nucleotide polymorphisms (SNPs) with genome-wide significance levels associated with exposure to TDFDs as instrumental variables. Inverse variance weighted (IVW) was employed as the primary method for Mendelian randomization (MR) analysis, supplemented by MR‒Egger, weighted median, simple mode and weighted mode methods. Sensitivity analyses were conducted to evaluate the robustness of the findings. The IVW method demonstrated an increased risk of OP in patients with TDFDs, including hyperthyroidism and hypothyroidism (TDFDs: OR = 1.11; 95% CI 1.09, 1.13; hypothyroidism: OR = 1.14; 95% CI 1.10, 1.17; hyperthyroidism: OR = 1.09; 95% CI 1.06, 1.12). These findings were supported by supplementary analysis, which revealed a positive correlation between TDFDs and the risk of OP. Multiple sensitivity analyses confirmed the absence of horizontal pleiotropy in the study, thus indicating the robustness of our results. The causal relationship between TDFDs and increased risk of OP implies the need for early bone mineral density (BMD) screening and proactive prevention and treatment strategies for individuals with TDFDs.

## Introduction

Osteoporosis is the most common metabolic bone disease and is characterized by a decrease in bone mass and changes in bone microstructure, resulting in decreased bone strength and an increased risk of fractures^1^. Fragility fractures of the hip and vertebra are the most severe consequences of osteoporosis and are associated with increased incidence and mortality rates^2,3^. Despite the availability of preventive and therapeutic medications, the incidence and associated costs of OP continue to rise globally. Osteoporosis is a significant public health concern worldwide, particularly in today's increasingly ageing population^4,5^. The development of osteoporosis is closely linked to endocrine disorders, such as diabetes^6,7^, abnormalities in lipid metabolism^8^, hyperprolactinemia^9^, Cushing's syndrome^10^, and thyroid dysfunction^11^.

Thyroid hormones play a crucial role in the body's homeostasis and metabolism^12,13^. They also exert crucial influences on skeletal growth and development, as well as accumulation of bone minerals, which serve as fundamental substances for maintaining optimal bone health^14^. TDFDs such as hyperthyroidism and hypothyroidism partially disrupt normal bone metabolism, thereby contributing to the risk factors associated with osteoporosis^11,15,16^. Hyperthyroidism can stimulate the activity of both osteoblasts and osteoclasts; however, due to the prevailing dominance of osteoclasts, bone resorption surpasses bone formation, leading to a decrease in bone mass and ultimately resulting in the development of osteoporosis^14,17^. Additionally, previous research has demonstrated that thyroid-stimulating hormone (TSH), which serves as a crucial regulatory factor for thyroid hormones, functions as an individual molecular switch that autonomously governs both bone formation and resorption^18^. Although existing evidence suggests a strong correlation between thyroid hormone dysfunction and osteoporosis, establishing a definitive causal link is challenging due to potential confounding factors.

Mendelian randomization (MR) employs genetic variants associated with exposure as instrumental variables in accordance with Mendel's second law to mitigate confounding factors and reverse causality. MR serves as a valuable tool for establishing causal associations and ranks second only to randomized controlled trials (RCTs) in terms of the level of evidence^19^. It is imperative to evaluate the genetic predisposition for thyroid dysfunction disorders, including hypothyroidism and hyperthyroidism, in relation to osteoporosis while investigating their potential direct causal impact on osteoporosis and associated fractures. Using large-scale GWAS data sources, we applied two-sample MR analysis^20–22^, which included the methods of “MR‒Egger”, “weighted median”, “inverse variance weighted”, “simple mode”, and “weighted mode”, to offer valuable clinical insights for the prevention, diagnosis and treatment of osteoporosis.

## Materials and methods

### Study design

**Figure 1:**
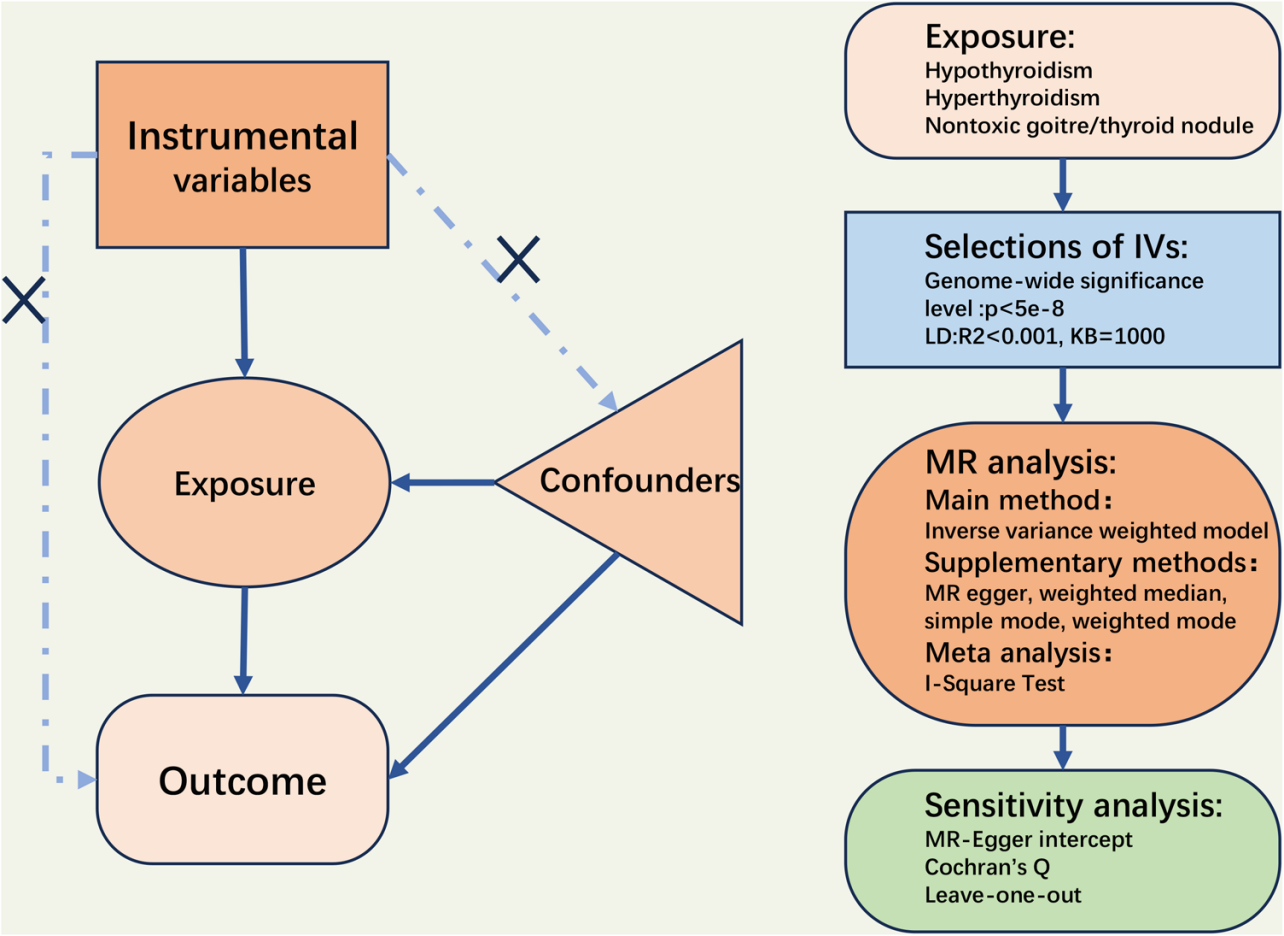
Study design and overview of MR.

In this study, we considered TDFDs as an exposure factor, we identified SNPs significantly associated with TDFDs as instrumental variables, and we evaluated the impact of these SNPs on OP outcomes using two-sample MR analysis. To ensure reliable selection of instrumental variables for two-sample MR analysis, three key assumptions were adhered to in this study: ① there is a significant correlation between instrumental variables and TDFDs; ② instrumental variables are not associated with confounders; and ③ instrumental variables solely influence outcomes through their association with TDFDs. However, testing assumptions 2 and 3 poses challenges due to their relevance to associations involving unknown confounders. Therefore, we employed the MR‒Egger regression coefficient estimation method to evaluate the presence of a horizontal pleiotropic effect and examined whether the intercept significantly deviated from zero. We adopted the IVW method as our primary approach and applied MR‒Egger, weighted median, simple mode and weighted mode methods as complementary methods to maintain accuracy and robustness. Then a series of sensitivity analyses were conducted, including Cochran's Q test, funnel plot, leave-one-out analysis, and Egger's intercept test to evaluate heterogeneity among single-SNPs and horizontal pleiotropy. Ultimately, a meta-analysis accompanied by the I-square testing of heterogeneity was conducted on the IVW results to assess the total impact of TDFDs on OP. An overview of the study design and the assumptions of the MR analysis are illustrated in Fig. 1.

### GWAS data sources

In this study, GWAS data on TDFTs and OP were acquired from The FinnGen biobank and were adjusted for age, sex, 10 principal components, and genotyping batch. More details about FinnGen are described in https://finngen.gitbook.io/documentation. In this study, we selected “autoimmune hyperthyroidism” and “Grave’s disease (strict definition)” as representative forms of hyperthyroidism and considered “hypothyroidism (strict autoimmune)” and “hypothyroidism (drug reimbursement)” as representative forms of hyperthyroidism. Additionally, we classified “nontoxic goitre/thyroid nodule” as dysfunction-free thyroid disease. We considered “osteoporosis”, “osteoporosis with pathological fracture”, and “postmenopausal osteoporosis with pathological fracture” as representative manifestations of osteoporosis. The sex ratio and age distribution are shown in Supplementary Fig. 1. At present, research shows that two-sample MR methods can be safely used for one-sample MR performed using data from large biobanks^23^. To assess potential overlap bias, we used an online tool (https://sb452.shinyapps.io/overlap/)^24^. The results showed that the bias of all groups was 0.000–0.001, and the Type I error rate was 0.05 (Supplementary Fig. 2). The study population was exclusively composed of individuals of European descent, ensuring the elimination of any potential bias caused by factors related to racial admixture. The details regarding the data sources utilized and the demographic profiles of TDFDs and OP are presented in Table 1.

**Table 1:** Data sources utilized in the present study.

Exposures or outcome	Abbreviation	Number of case	Number of control	Ancestry	Datasets in the GWAS
Grave’s disease (strict definition)	GV	3176	409,005	European	finngen_R10_E4_ GRAVES_STRICT
Autoimmune hyperthyroidism	AIHYPER	1991	305,175	European	finngen_R10_E4_ THYTOXGOITMULT
Hypothyroidism (drug reimbursement)	HYPOD	13,429	94,436	European	finngen_R10_ HYPOTHY_REIMB
Hypothyroidism (strict autoimmune)	AIHYPO	45,321	298,847	European	finngen_R10_E4_ HYTHY_AI_STRICT
Nontoxic goitre/thyroid nodule	NT	10,312	401,869	European	finngen_R10_E4_ NONTOXIC_THYROID
Osteoporosis	OP	8017	391,037	European	finngen_R10_M13_ OSTEOPOROSIS
Osteoporosis with pathological fracture	OF	1822	311,210	European	finngen_R10_OSTEOPOROSIS _FRACTURE_FG
Postmenopausal osteoporosis with pathological fracture	OFP	1486	228,601	European	finngen_R10_OSTPO PATFRCTURE_POSTEMENO

### Instrument variant selection

This study set the whole-genome significance criterion with a threshold of *p* < 5e–8. To address the issue of linkage disequilibrium (LD) for significant SNPs, the SNPs were clumped using PLINK1.9 with the following thresholds: R^2^ < 0.001 and window size < 10,000 kb^25^. Instrumental variables correlating significantly with TDFDs were identified, and their allele effects (β), standard errors (Se), and* p* values were recorded. The PhenoScanner v2 database was utilized to systematically search for and exclude confounding SNPs to meet the requirements of the second assumption^26^. Additionally, to assess the strength of the instrumental variables, we employed the following formula to calculate the F-statistic for each SNP: ${\text{F}}\, = \,{\text{R}}^{{2}} \left( {{\text{N}} - {\text{k}} - {1}} \right)/\left[ {{\text{k}}\left( {{1} - {\text{R}}^{{2}} } \right)} \right],$ where N represents the sample size, k denotes the number of instrumental variables, and R^2^ signifies the degree of exposure variation explained by each instrumental variable. The Formula R^2^ = β^2^ / [β^2^ + N × Se^2^] was used for calculation purposes. The strong correlation between IVs and TDFDs, as indicated by F > 10, ensures that the results of MR analysis are robust against weak instrumental bias^27^.

### MR analysis

The 'TwoSampleMR' package in R version 4.2.3 was used for conducting MR analysis in this study. Five different MR methods were utilized to evaluate the impact of TDFDs on OP: IVW, MR‒Egger, weighted median, simple mode and weighted mode methods. The IVW method utilizes the Wald ratio approach to estimate the exposure effect associated with each SNP, followed by weighted linear regression analysis. Given that the IVs satisfy the aforementioned assumptions, the IVW method typically demonstrates high accuracy^28^. Consequently, in this study, we primarily employed the IVW method as our main method. The MR‒Egger method can yield robust estimates that are not influenced by the effectiveness of instrumental variables (IVs) and can adjust for horizontal pleiotropy through regression slopes and intercepts^29,30^. However, the evaluation precision of this method is relatively low. Assuming a minimum of 50% IV effectiveness, the weighted median method can offer accurate and robust performance assessment^31^. We additionally integrated mode-based approaches, such as simple and weighted modes, to assess the causal impact of individual SNPs in forming clusters. The simple mode identifies the largest cluster for estimating the causal effect of SNPs, whereas the weighted mode assigns specific weights to each SNP^32,33^. Bonferroni correction was applied to the results of multiple correlation tests, with a significance level set at *p* < 0.0042 (*p* < 0.05/3/4)^34^; *p* values between 0.0042 and 0.05 were considered indicative of potential causation.

### Sensitivity analyses

MR‒Egger regression and funnel plots can be employed for detecting pleiotropy. MR Egger regression’s intercept term facilitates the evaluation and adjustment of pleiotropy levels pertaining to IVs^29^. Furthermore, an asymmetrical funnel plot can be utilized as a test for horizontal pleiotropy^27^. Additionally, the Cochran’s Q method and leave-one-out method were utilized to assess heterogeneity among single-SNPs. A *p*-value less than 0.05 for the Cochran’s Q test indicates the presence of heterogeneity among single-SNPs, necessitating an adjustment of IVW to a random effects model. Furthermore, in the leave-one-out method analysis, each SNP was sequentially removed, and the remaining SNPs were subjected to IVW analysis to identify any outliers with a significant impact on the results^35^. Finally, scatter plots and forest plots were generated to display the outcomes of the MR analysis.

## Results

### Hypothyroidism and osteoporosis

**Figure 2:**
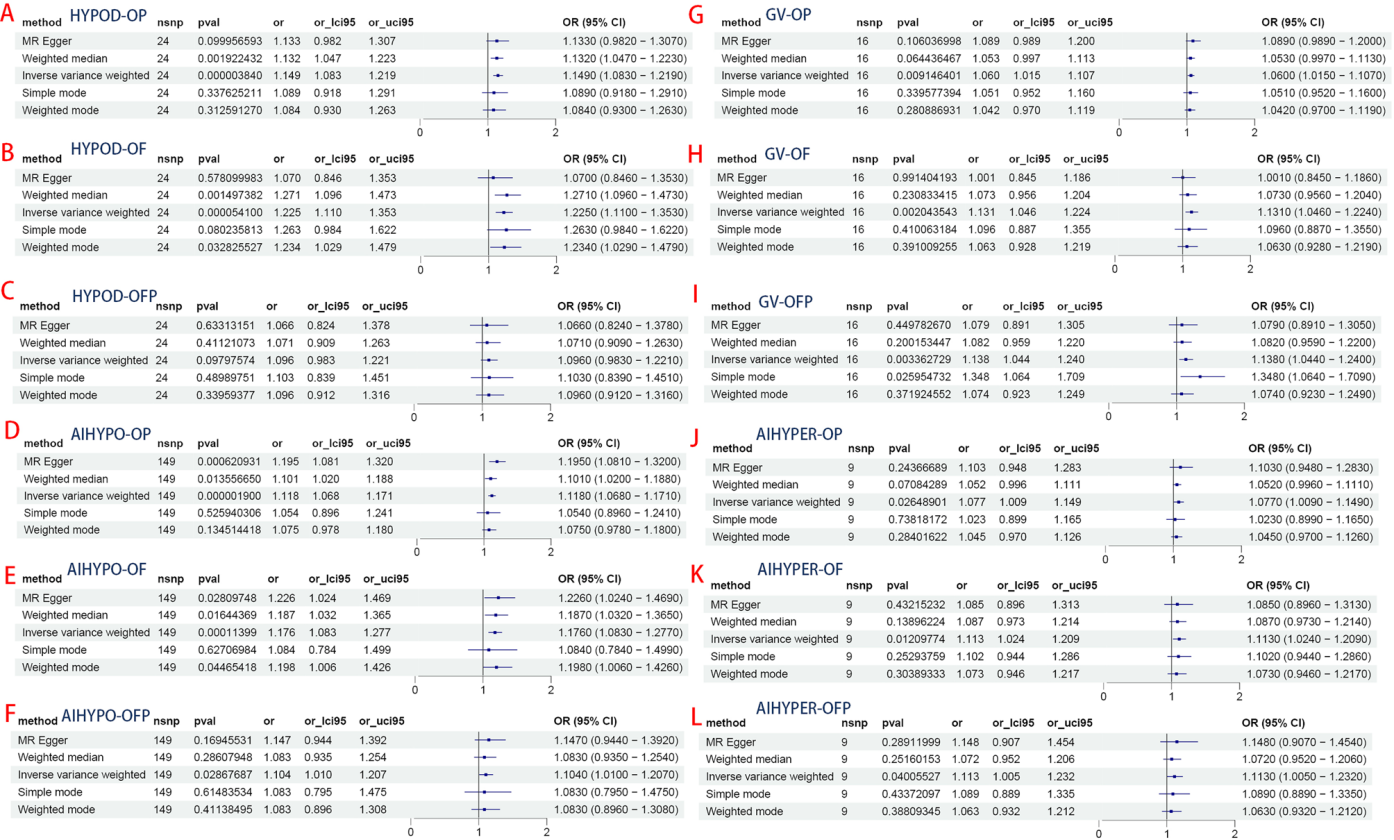
Analysis findings obtained from five Mendelian randomization methods.

**Figure 3:**
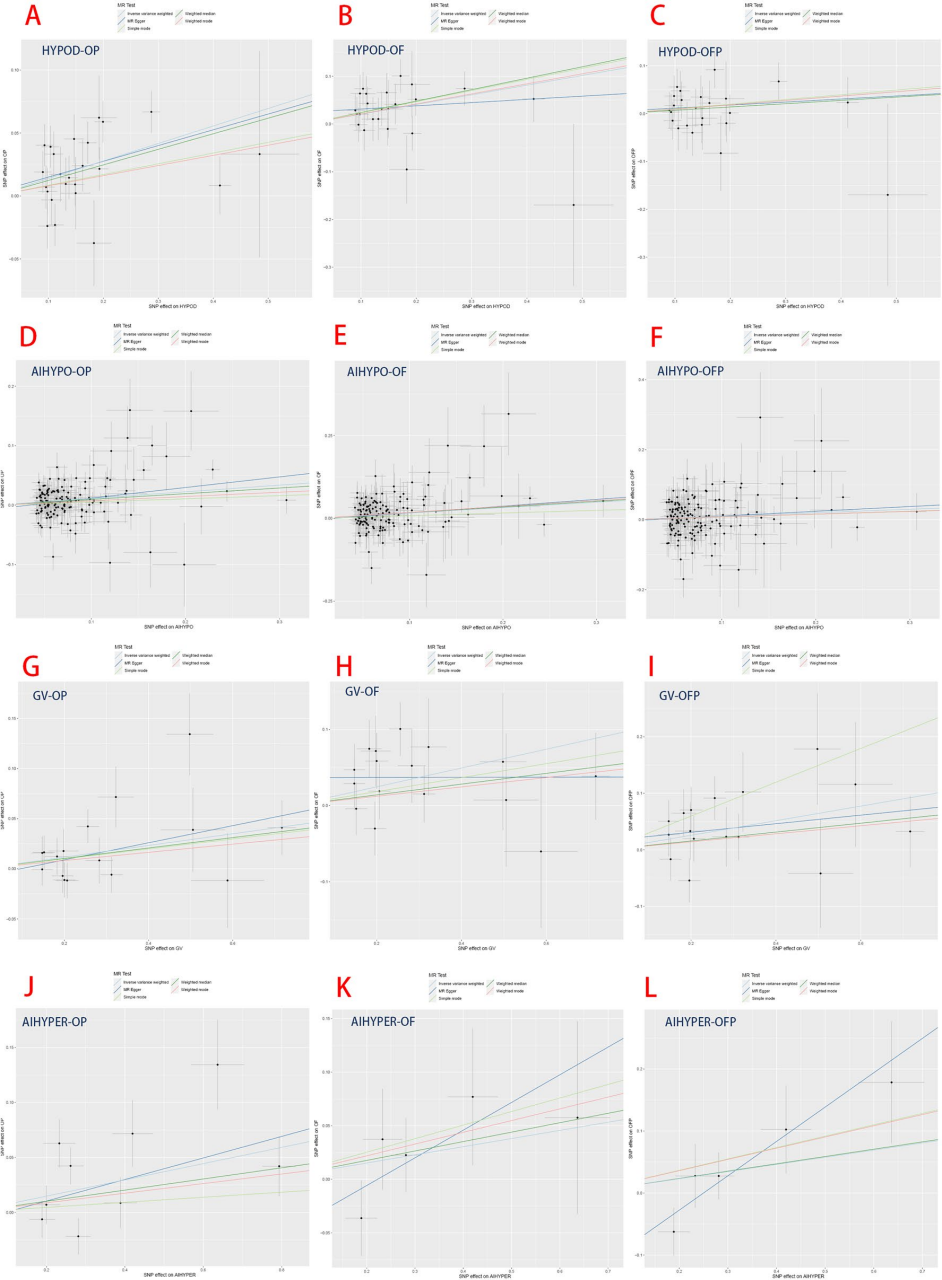
MR test scatter plot of the five methods.

**Figure 4:**
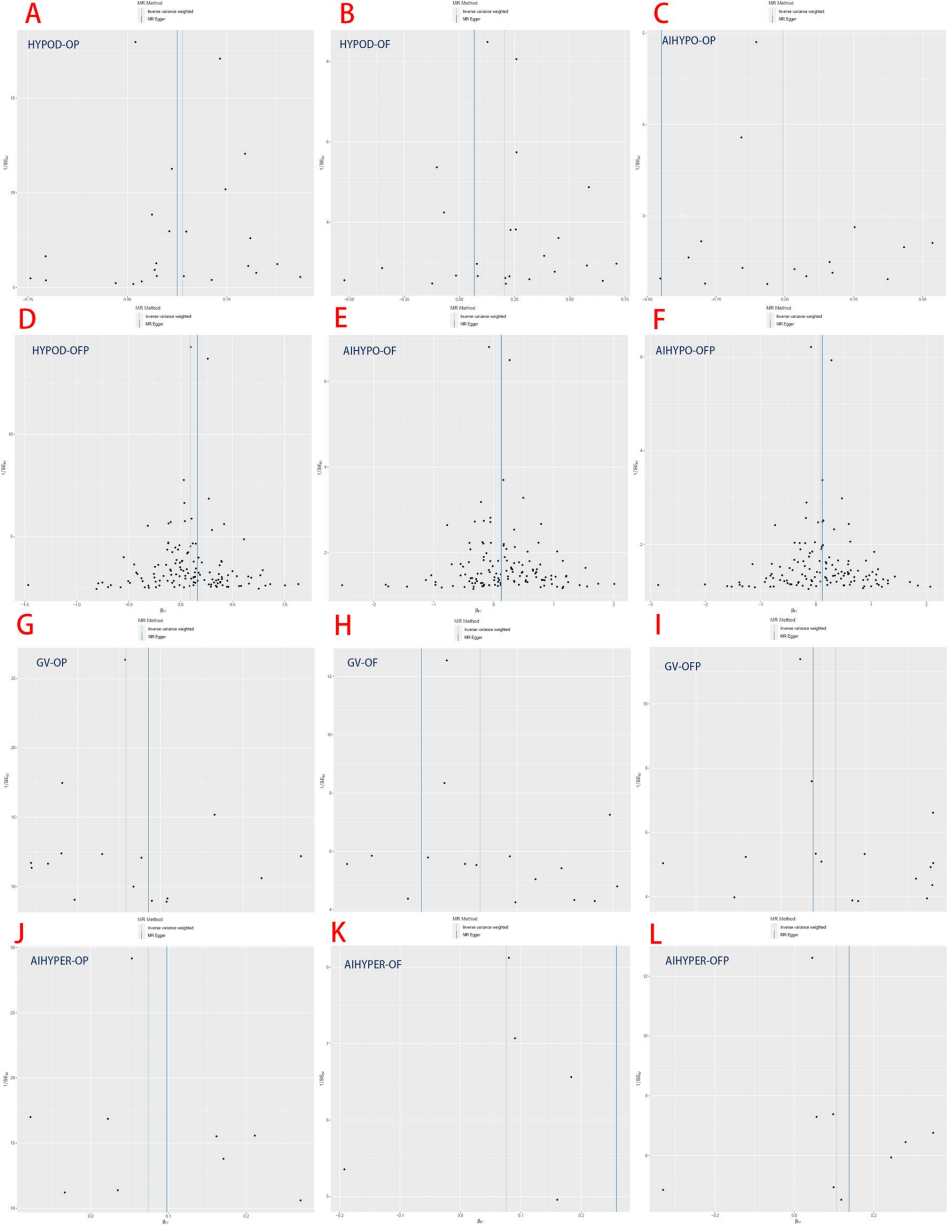
MR funnel plot of the IVW and MR‒Egger methods.

**Figure 5:**
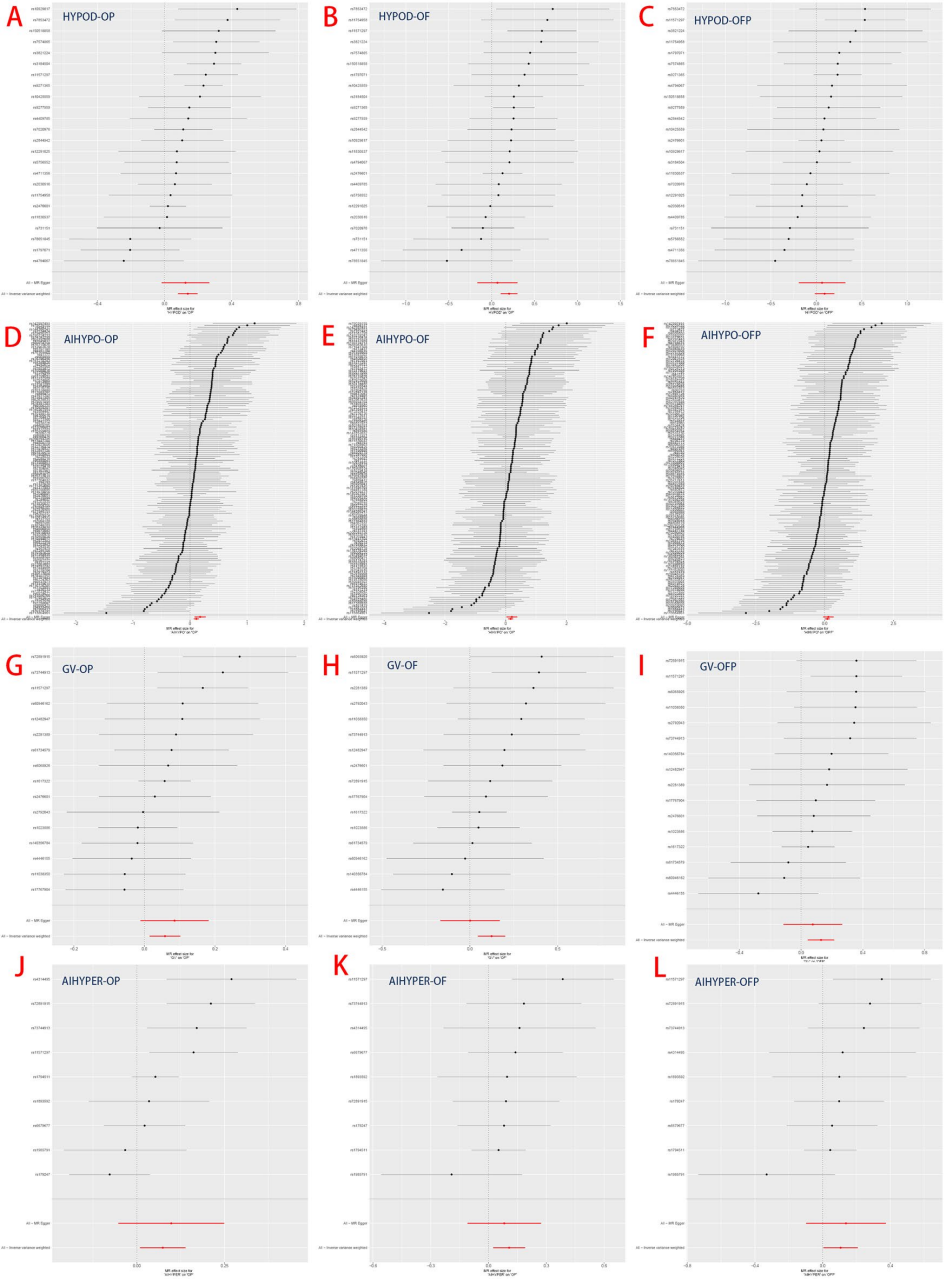
Forest plot of the MR sensitivity analysis.

**Figure 6:**
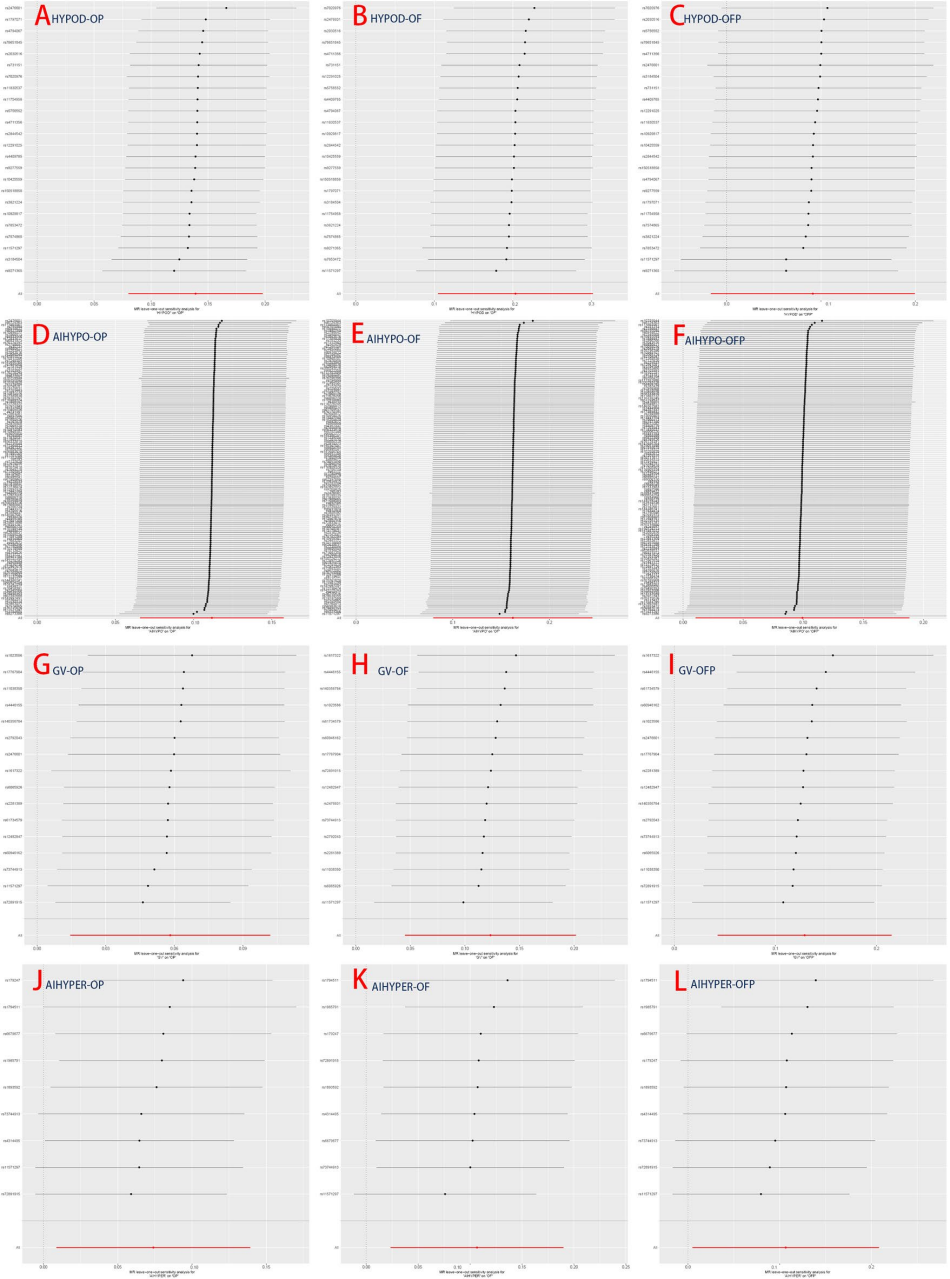
Forest plot of the MR leave-one-out sensitivity analysis.

**Figure 7:**
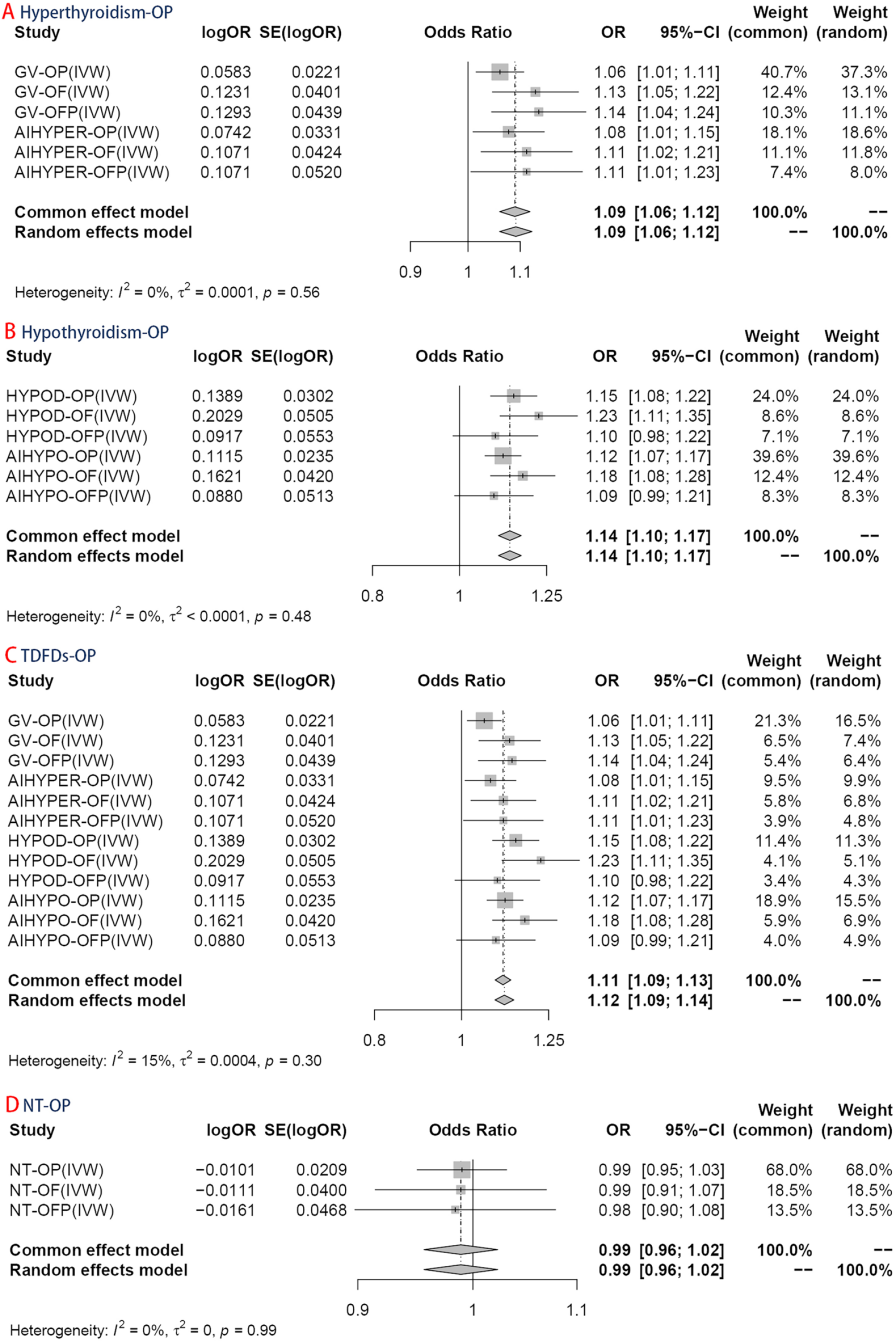
Results of the meta-analysis of TDFDs and NTs.

The results demonstrated a significant causal association between hypothyroidism and the risk of osteoporosis in individuals of European ancestry, employing the IVW method with four significant associations and one potential causal factor (HYPOD-OP: OR = 1.149, 95% CI 1.083, 1.219, *p* < 0.0042; HYPOD-OF: OR = 1.225, 95% CI 1.110, 1.353, *p* < 0.0042; HYPOD-OFP: OR = 1.096, 95% CI 0.983, 1.221, *p* = 0.0980; AIHYPO-OP: OR = 1.118, 95% CI 1.068, 1.171, *p* < 0.0042; AIHYPO-OF: OR = 1.176, 95% CI 1.083, 1.277, *p* < 0.0042; AIHYPO-OFP: OR = 1.104, 95% CI 1.010, 1.207, *p* < 0.05) (Fig. 2A–F). The OR values obtained from the MR‒Egger, weighted median, simple mode, and weighted mode methods were consistent with those obtained from the IVW method, indicating the robustness of the results (Fig. 3A–F). The meta-analysis technique was used to integrate and analyse the impact of hypothyroidism on osteoporosis (common-effect model: OR = 1.14; 95% CI 1.10, 1.17; I-square test *p* = 0.48) (Fig. 7B). According to Cochran’s Q test, *p* > 0.05 for IVW and MR‒Egger indicated no evidence of heterogeneity among single-SNPs. As we found heterogeneity among single-SNPs in HYPOD-OP (IVW: Q = 36.37, *p* = 0.04) and AIHYPO-OP (IVW: Q = 180.95, *p* < 0.01), random-effects IVW was applied (Table 2). Additionally, MR‒Egger regression results suggested no evidence of horizontal pleiotropy in this study (*p* > 0.05) (Table 2). Funnel plot analysis revealed a symmetrical scatter distribution of causal effects when SNPs were used as instrumental variables (IVs), further supporting the findings based on MR‒Egger regression (Fig. 4A–F). Moreover, sensitivity analysis demonstrated that excluding each SNP individually did not significantly alter the IVW analysis results compared to including all SNPs, and no influential SNPs affecting the estimated causal relationship between hypothyroidism and OP were identified, indicating the robustness of our findings (Figs. 5A–F and 6A–F).

**Table 2:** MR sensitivity analyses of TDFDs and OP.

Exposures	Outcomes	nSNPs	Cochran’s heterogeneity test		Pleiotropy test	
			Single-SNP IVW		MR-Egger intercept	
			Q	*p* val	Intercept	*p* val
Sensitivity analysis of thyroid dysfunction on hypothyroidism						
GV	OP	16	20.72	0.15	− 0.0082	0.55
GV	OF	16	15.04	0.57	0.0368	0.13
GV	OFP	16	14.98	0.45	0.016	0.55
AIHYPER	OP	9	21.91	< 0.01*	− 0.0093	0.74
AIHYPER	OF	9	7.85	0.45	0.0096	0.78
AIHYPER	OFP	9	9.88	0.27	− 0.0121	0.78
HYPOD	OP	24	36.37	0.04	0.0026	0.84
HYPOD	OF	24	21.79	0.53	0.0244	0.22
HYPOD	OFP	24	16.08	0.81	0.005	0.82
AIHYPO	OP	149	180.95	< 0.01*	− 0.0058	0.21
AIHYPO	OF	149	152.6	0.38	− 0.0038	0.61
AIHYPO	OFP	149	142.61	0.61	− 0.0034	0.67
NT	OP	46	61.93	0.04	− 0.007	0.42
NT	OF	46	52.12	0.22	0.0014	0.93
NT	OFP	46	58.84	0.08	− 0.0098	0.62

*p val < 0.05.

### Hyperthyroidism and osteoporosis

There was a significant causal relationship between hyperthyroidism and osteoporosis according to the IVW method, with two significant associations and four potential causations (GV-OP: OR = 1.060, 95% CI 1.015, 1.107, *p* < 0.05; GV-OF: OR = 1.131, 95% CI 1.046, 1.353, *p* < 0.0042; GV-OFP: OR = 1.138, 95% CI 1.044, 1.240, *p* < 0.0042; AIHYPER-OP: OR = 1.077, 95% CI 1.009, 1.149, *p* < 0.05; AIHYPER-OF: OR = 1.113, 95% CI 1.024, 1.209, *p* < 0.05; AIHYPER-OFP: OR = 1.113, 95% CI 1.005, 1.232, *p* < 0.05) (Fig. 2G–L). The consistent results of the aforementioned five methods indicated a causal relationship between hyperthyroidism and osteoporosis (Fig. 3G–L). The results of the meta-analysis revealed the total effect of hyperthyroidism on osteoporosis (common effect model: OR = 1.09, 95% CI 1.06, 1.12, I-square test *p* = 0.56) (Fig. 7A). Random-effects IVW was applied, as Cochran’s Q test revealed heterogeneity among single-SNPs in AIHYPER-OP (IVW: Q = 21.91, *p* < 0.01) (Table 2). MR‒Egger regression and funnel plot analyses indicated no obvious horizontal pleiotropy in this study (*p* > 0.05) (Table 2). The leave-one-off method also demonstrated the absence of significant heterogeneity among single-SNPs (Figs. 5G–L and 6G–L).

### TDFDs and osteoporosis

**Figure 8:**
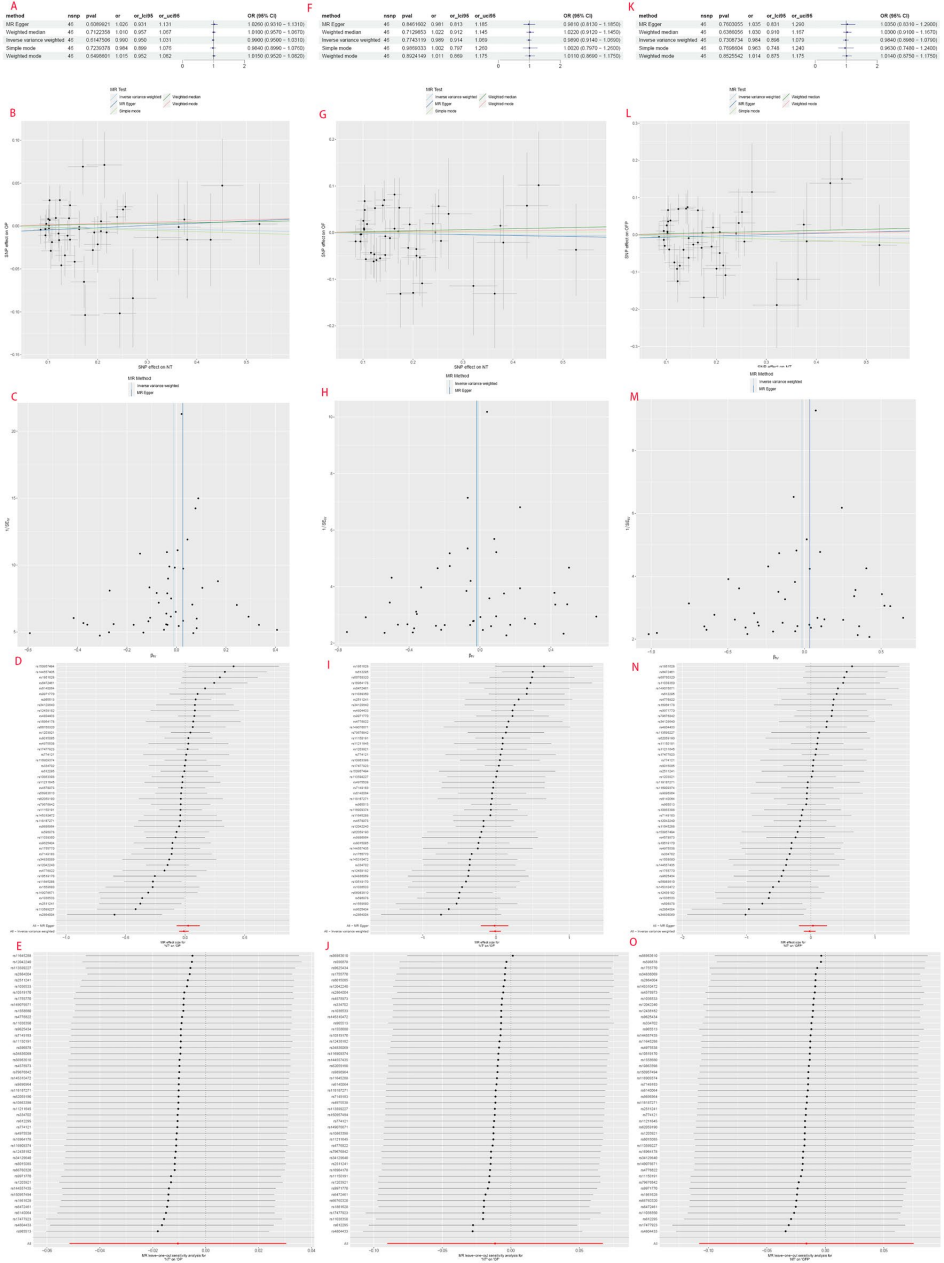
Mendelian randomization results, scatter plot, funnel plot, forest plot and the result of the leave-one-out method of NT.

The IVW results for hyperthyroidism and hypothyroidism were merged, as these two forms are commonly observed in TDFDs (common effect model: OR = 1.11, 95% CI 1.09, 1.13; I-square test *p* = 0.30) (Fig. 7C). The results demonstrated a significant association between genetic prediction of TDFDs and elevated susceptibility to osteoporosis. The present study also employed nontoxic goitre/thyroid nodule (NT) as an exposure factor to conduct MR analysis on osteoporosis, aiming to investigate the causal relationship between nonfunctional thyroid disease and estimated osteoporosis as well as concurrent fractures. The outcome we achieved was negative according to the IVW method (META analysis: OR = 0.99, 95% CI 0.96, 1.02, I-square test *p* = 0.99; NT-OP: OR = 0.990, 95% CI 0.950, 1.031, *p* = 0.615; NT-OF: OR = 0.989, 95% CI 0.914, 1.069, *p* = 0.774; NT-OFP: OR = 0.984, 95% CI 0.898, 1.079; *p* = 0.731), which is consistent with the findings of the other four methods (Figs. 7D and 8).

## Discussion

There is a substantial worldwide medical and economic burden on society due to the ageing population, given that ageing serves as a risk factor for several prevalent diseases globally, including neurodegenerative disorders, cancer, cardiovascular ailments, and metabolic conditions^36,37^. Ying et al. identified a comprehensive landscape of CpG sites with potential causal links to lifespan and healthspan, including the CpG site associated with head bone mineral density^38^. Osteoporosis and TDFDs are recognized as age-related conditions with a high prevalence among the elderly population^5,39^. In recent years, numerous researchers have conducted studies focusing on targets for longevity and age-related diseases^40–42^. Previous studies have explored associations between thyroid hormone-related characteristics such as hyperthyroidism, TSH, and TSH receptors and bone density in relation to TDFDs and osteoporosis, but the results seem to be inconsistent^43–46^. The present study included five distinct clinical phenotypes of thyroid disorders (including hyperthyroidism, hypothyroidism, and nonfunctional thyroid disease) as well as three clinical phenotypes of osteoporosis (including osteoporosis and fracture), providing a more comprehensive investigation into the causal association between TDFDs and osteoporosis. We employed MR analysis to investigate the association between TDFDs and osteoporosis using genetic data obtained from publicly available databases. To minimize potential confounding factors arising from population heterogeneity, we restricted the MR study population to individuals of European ancestry. MR analysis consistently demonstrated a correlation between TDFDs and a high risk of OP and related fractures, as evidenced by both IVW results and other supplementary methods. Furthermore, multiple sensitivity analyses were conducted to ensure the robustness and accuracy of the findings.

Osteoporosis, the most common bone metabolic disease, is closely related to the endocrine system^47^. The bone remodelling cycle is regulated by systemic hormones and local factors, and its process includes activation, absorption, reversal and formation stages. Enhanced osteoclast activity and/or reduced osteoblast activity result in net bone loss, thereby increasing the risk of developing osteoporosis^48^. Thyroid hormones play an important role in bone metabolism, including bone formation and resorption processes^11,14^. Additionally, close associations between thyroid hormones and bone density, osteoporosis, and osteoporotic fractures have been consistently observed in clinical practice^49,50^.

Thyroid hormones exert significant effects on bone development, linear growth, and maintenance of adult bone mass and strength^14,51^. In children, T3 interacts with TRα in chondrocytes and osteoblasts to regulate intramembrane and endochondral ossification, controlling the rate of linear growth as well as bone maturation and mineralization^52^. Research has demonstrated that maintaining normal thyroid function during growth and adolescence is crucial for establishing optimal peak bone mass and strength in early adulthood^53^. Conversely, in adults, while thyroid hormone synthesis metabolism stimulates bone growth and mineralization in juvenile bones, T3 plays a catabolic role by promoting bone loss^52^.

The rate of bone turnover is accelerated in individuals with hyperthyroidism, consistent with the findings of previous studies^14,51^. This phenomenon can be explained from two perspectives. First, elevated thyroid hormone levels can stimulate bone formation and osteoclast differentiation by binding to the thyroid hormone receptor α1 on bone tissue, ultimately leading to an increased rate of bone turnover^14^. Second, TSH plays a crucial role as an influencing factor that inhibits osteoclast maturation and reduces osteoblast formation, thereby safeguarding the integrity of the skeletal system. Previous research has established a pivotal role for TSH as a singular molecular switch in autonomous regulation of both bone formation and resorption by attenuating JNK/c-jun and NFκB signalling in osteoclasts and downregulating Wnt (LRP-5) and VEGF (Flk) signalling in osteoblasts^18^. Moreover, impaired thyroid hormone sensitivity is associated with osteoporosis and fractures independent of other traditional risk factors^54^. Although there is research demonstrating the absence of a causal association between an increased genetic predisposition for hyperthyroidism and the risk of low bone mineral density^45^, the present study identified Grave’s disease and autoimmune hyperthyroidism as potential risk factors, and osteoporosis and osteoporotic fractures were the outcomes. These findings highlight the causal association of hyperthyroidism with an increased risk of developing osteoporosis and related fractures.

Hypothyroidism results in a decreased metabolic rate throughout the body, and its symptoms lack specificity. Hypothyroidism has a relatively high incidence, ranging from 0.2–5.3% for overt hypothyroidism and 3–10% for subclinical hypothyroidism^55,56^. Because it is difficult to distinguish hypothyroidism from other diseases based solely on symptoms, diagnosis depends on measurement of TSH and free thyroxine levels^57^. A reduction in osteoclast bone resorption and osteoblast activity leads to a decrease in bone metabolism. Moreover, acceleration of bone loss has been demonstrated in patients with hypothyroidism^46^. Relevant studies have yielded inconclusive findings regarding the association between subclinical hypothyroidism and osteoporosis as well as fractures^58,59^. To the best of our knowledge, there is currently a dearth of MR studies investigating the association between hypothyroidism and the risk of osteoporosis and fractures. In this study, we found that hypothyroidism increases the risk of osteoporosis and fractures. Moreover, because of the typical phenotypes of TDFDs, we found that both hyperthyroidism and hypothyroidism increase the risk of osteoporosis and related fractures.

In this study, two-sample MR method was employed to evaluate the causal effect of TDFDs on OP from a genetic perspective, which has certain advantages. First, the genetic variation in MR analysis occurs at conception and is not affected by external factors. It is the closest approach to randomized controlled trials, which saves time and cost. Second, MR studies can avoid some limitations of observational studies, including reverse causality and potential confounding factors. In addition, a large sample size of included studies and instrumental variables significantly related to TDFDs, as well as multiple sensitivity analysis, were used, which enhances the robustness of the results. However, there are also some shortcomings in the study. Firstly, because it is based on MR analysis at the summary data level, it is impossible to explore the potential nonlinear effects of TDFDs on OP. Further studies are necessary to verify the nature of these relationships. Secondly, we solely employed statistical methods without incorporating biological connections in the selection of instrument variants, which may introduce potential biases. At last, considering that allele frequency in heterogeneous ethnic populations may lead to bias in genetic studies, MR analysis was limited to homogeneous ethnic populations to avoid potential confusion from more heterogeneous populations; interpretation of the results and application to other ethnic populations are limited.

## Conclusion

In conclusion, the MR method was used to investigate the causal association between TDFDs and OP from a genetic perspective. These findings have important implications for guiding clinical practice. Therefore, early screening of bone density and proactive measures for preventing and treating osteoporosis in patients with TDFDs are recommended.

### Supplementary Information

Supplementary Figure 1.

Supplementary Figure 2.

## Data Availability

The GWAS data on TDFTs and OP were acquired from The FinnGen Biobank (www.finngen.fi) in this study (Table 1).

## References

[1] Ensrud KE, Crandall CJ (2017). Osteoporosis. Ann. Intern. Med..

[2] Guzon-Illescas O (2019). Mortality after osteoporotic hip fracture: Incidence, trends, and associated factors. J. Orthop. Surg. Res..

[3] Barton DW, Behrend CJ, Carmouche JJ (2019). Rates of osteoporosis screening and treatment following vertebral fracture. Spine J..

[4] Anam AK, Insogna K (2021). Update on osteoporosis screening and management. Med. Clin. North Am..

[5] Harvey N, Dennison E, Cooper C (2010). Osteoporosis: Impact on health and economics. Nat. Rev. Rheumatol..

[6] Parizad N (2019). The prevalence of osteoporosis among Iranian postmenopausal women with type 2 diabetes: A systematic review and meta-analysis. Diabetes Metab. Syndrome.

[7] Schwartz AV (2022). Risk factors for lower bone mineral density in older adults with type 1 diabetes: A cross-sectional study. Lancet Diabetes Endocrinol..

[8] Zhang Z, Duan Y, Huo J (2023). Lipid metabolism, methylation aberrant, and osteoporosis: A multi-omics study based on mendelian randomization. Calcif. Tissue Int..

[9] Clapham E (2020). Exposure to risperidone versus other antipsychotics and risk of osteoporosis-related fractures: a population-based study. Acta Psychiatr. Scand..

[10] Wang D (2022). Relationship between osteoporosis and Cushing syndrome based on bioinformatics. Medicine.

[11] Delitala AP, Scuteri A, Doria C (2020). Thyroid hormone diseases and osteoporosis. J. Clin. Med..

[12] Mullur R, Liu Y-Y, Brent GA (2014). Thyroid hormone regulation of metabolism. Physiol. Rev..

[13] Wenzek C (2022). The interplay of thyroid hormones and the immune system—We stand and why we need to know about it. Eur. J. Endocrinol..

[14] Bassett JHD, Williams GR (2016). Role of thyroid hormones in skeletal development and bone maintenance. Endocr. Rev..

[15] Bel Lassen P (2019). Graves' disease, multinodular goiter and subclinical hyperthyroidism. Ann. D'endocrinol..

[16] Xu N (2020). Effect of subclinical hyperthyroidism on osteoporosis: A meta-analysis of cohort studies. Endocrine.

[17] Mosekilde L, Eriksen EF, Charles P (1990). Effects of thyroid hormones on bone and mineral metabolism. Endocrinol. Metab. Clin. North Am..

[18] Abe E (2003). TSH is a negative regulator of skeletal remodeling. Cell.

[19] Emdin CA, Khera AV, Kathiresan S (2017). Mendelian randomization. JAMA.

[20] Jones HJ (2021). Associations between plasma fatty acid concentrations and schizophrenia: A two-sample Mendelian randomisation study. Lancet Psychiatry.

[21] Kappelmann N (2021). Dissecting the association between inflammation, metabolic dysregulation, and specific depressive symptoms: A genetic correlation and 2-sample Mendelian randomization study. JAMA Psychiatry.

[22] Hartwig FP (2016). Two-sample Mendelian randomization: Avoiding the downsides of a powerful, widely applicable but potentially fallible technique. Int. J. Epidemiol..

[23] Minelli C (2021). The use of two-sample methods for Mendelian randomization analyses on single large datasets. Int. J. Epidemiol..

[24] Burgess S, Davies NM, Thompson SG (2016). Bias due to participant overlap in two-sample Mendelian randomization. Genet. Epidemiol..

[25] Purcell S (2007). PLINK: A tool set for whole-genome association and population-based linkage analyses. Am. J. Hum. Genet..

[26] Staley JR (2016). PhenoScanner: A database of human genotype-phenotype associations. Bioinformatics (Oxford, England).

[27] Hemani G (2018). The MR-Base platform supports systematic causal inference across the human phenome. ELife.

[28] Burgess S, Butterworth A, Thompson SG (2013). Mendelian randomization analysis with multiple genetic variants using summarized data. Genet. Epidemiol..

[29] Bowden J, Davey Smith G, Burgess S (2015). Mendelian randomization with invalid instruments: effect estimation and bias detection through Egger regression. Int. J. Epidemiol..

[30] Burgess S, Thompson SG (2017). Interpreting findings from Mendelian randomization using the MR-Egger method. Eur. J. Epidemiol..

[31] Bowden J (2016). Consistent estimation in Mendelian randomization with some invalid instruments using a weighted median estimator. Genet. Epidemiol..

[32] Hartwig FP, Davey Smith G, Bowden J (2017). Robust inference in summary data Mendelian randomization via the zero modal pleiotropy assumption. Int. J. Epidemiol..

[33] Walker VM (2019). Using the MR-Base platform to investigate risk factors and drug targets for thousands of phenotypes. Wellcome Open Res..

[34] Curtin F, Schulz P (1998). Multiple correlations and Bonferroni's correction. Biol. Psychiatry.

[35] Xiao G (2022). Causality of genetically determined metabolites on anxiety disorders: A two-sample Mendelian randomization study. J. Transl. Med..

[36] Armanios M (2015). Translational strategies in aging and age-related disease. Nat. Med..

[37] Fang EF (2020). A research agenda for ageing in China in the 21st century (2nd edition): Focusing on basic and translational research, long-term care, policy and social networks. Ageing Res. Rev..

[38] Ying K (2024). Causality-enriched epigenetic age uncouples damage and adaptation. Nat. Aging.

[39] Mammen JSR (2023). Thyroid and aging. Endocrinol. Metab. Clin. North Am..

[40] Liu X (2023). Mendelian randomization analyses reveal causal relationships between the human microbiome and longevity. Sci. Rep..

[41] Mavromatis LA (2023). Multi-omic underpinnings of epigenetic aging and human longevity. Nat. Commun..

[42] Perrot N (2021). A trans-omic Mendelian randomization study of parental lifespan uncovers novel aging biology and therapeutic candidates for chronic diseases. Aging Cell.

[43] van Vliet NA (2018). Thyroid stimulating hormone and bone mineral density: evidence from a two-sample mendelian randomization study and a candidate gene association study. J. Bone Min. Res..

[44] Soto-Pedre E (2021). Evidence of a causal relationship between serum thyroid-stimulating hormone and osteoporotic bone fractures. Eur. Thyroid J..

[45] Deshmukh H (2021). Hyperthyroidism and bone mineral density: Dissecting the causal association with Mendelian randomization analysis. Clin. Endocrinol..

[46] Stall GM (1990). Accelerated bone loss in hypothyroid patients overtreated with L-thyroxine. Ann. Intern. Med..

[47] Rosen CJ (1997). Endocrine disorders and osteoporosis. Curr. Opin. Rheumatol..

[48] Parfitt AM (2000). Structural and cellular changes during bone growth in healthy children. Bone.

[49] SeyedAlinaghi S (2023). The relationship of hip fracture and thyroid disorders: A systematic review. Front. Endocrinol..

[50] Greenspan SL, Greenspan FS (1999). The effect of thyroid hormone on skeletal integrity. Ann. Intern. Med..

[51] Williams GR, Bassett JHD (2018). Thyroid diseases and bone health. J. Endocrinol. Investig..

[52] O'Shea PJ (2006). Characterization of skeletal phenotypes of TRalpha1 and TRbeta mutant mice: Implications for tissue thyroid status and T3 target gene expression. Nucl. Recept. Signal..

[53] Rivkees SA, Bode HH, Crawford JD (1988). Long-term growth in juvenile acquired hypothyroidism: The failure to achieve normal adult stature. New Engl. J. Med..

[54] Liu C (2023). Impaired sensitivity to thyroid hormone correlates to osteoporosis and fractures in euthyroid individuals. J. Endocrinol. Investig..

[55] Chaker L (2017). Hypothyroidism. Lancet (London, England).

[56] Vanderpump MPJ, Tunbridge WMG (2002). Epidemiology and prevention of clinical and subclinical hypothyroidism. Thyroid.

[57] Chaker L (2022). Hypothyroidism. Nat. Rev. Disease Primers.

[58] Lee K (2020). Subclinical thyroid dysfunction, bone mineral density, and osteoporosis in a middle-aged Korean population. Osteoporos. Int..

[59] Siru R (2018). Subclinical thyroid dysfunction and circulating thyroid hormones are not associated with bone turnover markers or incident hip fracture in older men. Clin. Endocrinol..

