# Supplementary material for: Investigating the causal relationship between thyroid dysfunction diseases and osteoporosis: a two-sample Mendelian randomization analysis
Source: Sci Rep. 2024 Jun 4;14:12784. doi: 10.1038/s41598-024-62854-x (PMC11150446; doi:10.1038/s41598-024-62854-x)

Supplementary Figure 1: The sex ratio and age distribution of each phenotype.

A. Graves disease, strict definition

Key figures

	All	Female	Male
? Number of individuals	3176	2689	487
? Unadjusted prevalence (%)	0.77	1.17	0.27
? Mean age at first event (years)	49.14	48.45	52.95

Age distribution of first events

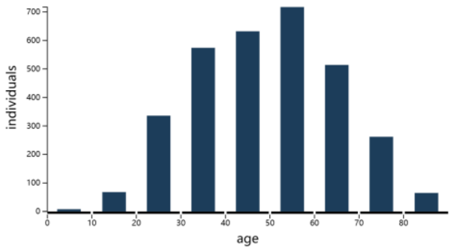

B. Autoimmune hyperthyroidism

Key figures

	All	Female	Male
? Number of individuals	1991	1647	344
? Unadjusted prevalence (%)	0.48	0.72	0.19
? Mean age at first event (years)	49.77	48.67	55.03

Age distribution of first events

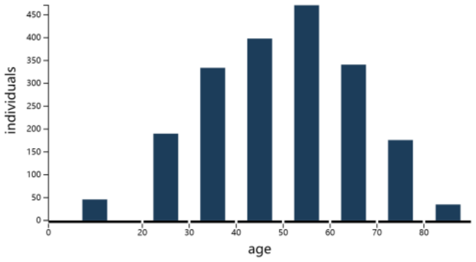

C. Hypothyroidism (drug reimbursement)

Key figures

	All	Female	Male
? Number of individuals	13428	11093	2335
? Unadjusted prevalence (%)	3.26	4.82	1.28
? Mean age at first event (years)	46.95	46.02	51.35

Age distribution of first events

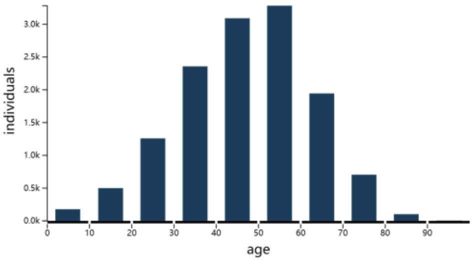

D. Hypothyroidism (strict autoimmune)

Key figures

	All	Female	Male
Number of individuals	44803	35884	8919
Unadjusted prevalence (%)	10.87	15.59	4.91
Mean age at first event (years)	51.54	49.70	58.95

Age distribution of first events

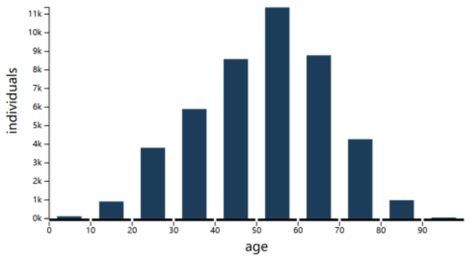

E. Nontoxic goitre/thyroid nodule

Key figures

	All	Female	Male
Number of individuals	10312	8568	1744
Unadjusted prevalence (%)	2.50	3.72	0.96
Mean age at first event (years)	51.76	50.47	58.14

Age distribution of first events

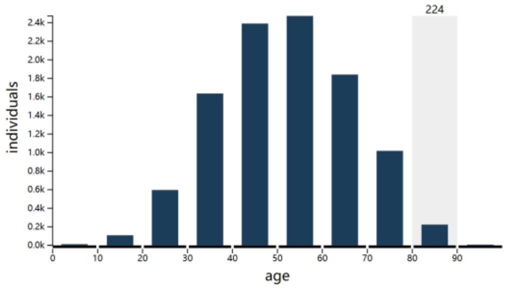

F. Osteoporosis

Key figures

	All	Female	Male
Number of individuals	8017	6084	1933
Unadjusted prevalence (%)	1.95	2.64	1.06
Mean age at first event (years)	67.42	67.97	65.68

Age distribution of first events

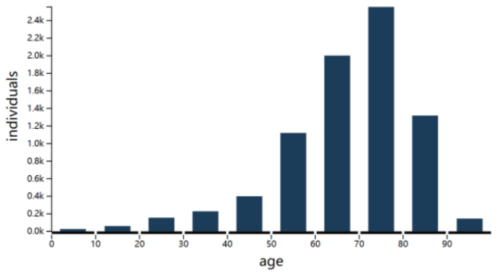

G. Osteoporosis with pathological fracture

Key figures

	All	Female	Male
Number of individuals	1822	1709	113
Unadjusted prevalence (%)	0.44	0.74	0.06
Mean age at first event (years)	70.95	71.33	65.20

Age distribution of first events

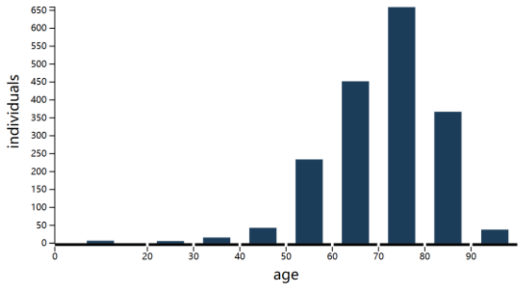

H. Postmenopausal osteoporosis with pathological fracture

Key figures

	All	Female	Male
Number of individuals	1486	1486	-
Unadjusted prevalence (%)	0.36	0.65	-
Mean age at first event (years)	72.37	72.37	-

Age distribution of first events

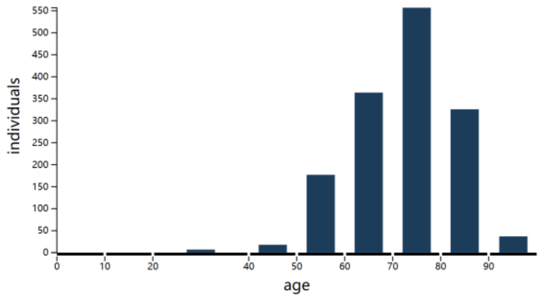

Supplement: Supplementary file 1 — Supplementary Figure 1. [file 41598_2024_62854_MOESM1_ESM.pdf]
