# Supplementary material for: Investigating the causal relationship between thyroid dysfunction diseases and osteoporosis: a two-sample Mendelian randomization analysis
Source: Sci Rep. 2024 Jun 4;14:12784. doi: 10.1038/s41598-024-62854-x (PMC11150446; doi:10.1038/s41598-024-62854-x)
Supplement: Supplementary file 2 — Supplementary Figure 2. [file 41598_2024_62854_MOESM2_ESM.pdf]

Supplementary Figure 2: The bias values and the Type I error rate of the overlap.

A. Bias and Type 1 error rate for Mendelian randomization with sample overlap of GV-OP

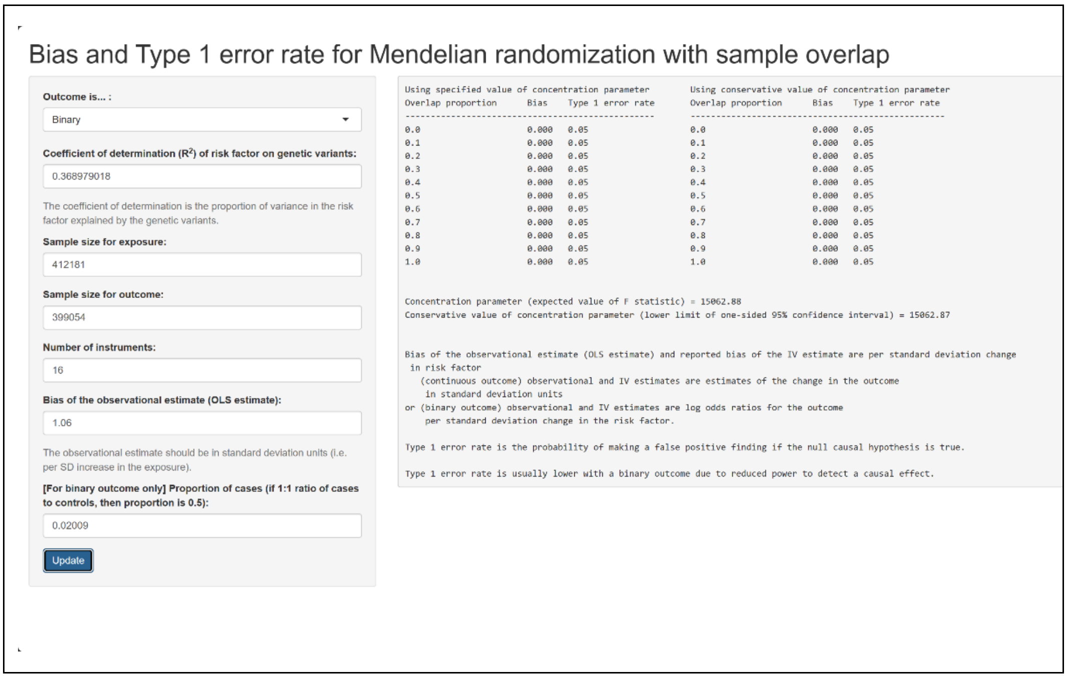

B. Bias and Type 1 error rate for Mendelian randomization with sample overlap of GV-OF

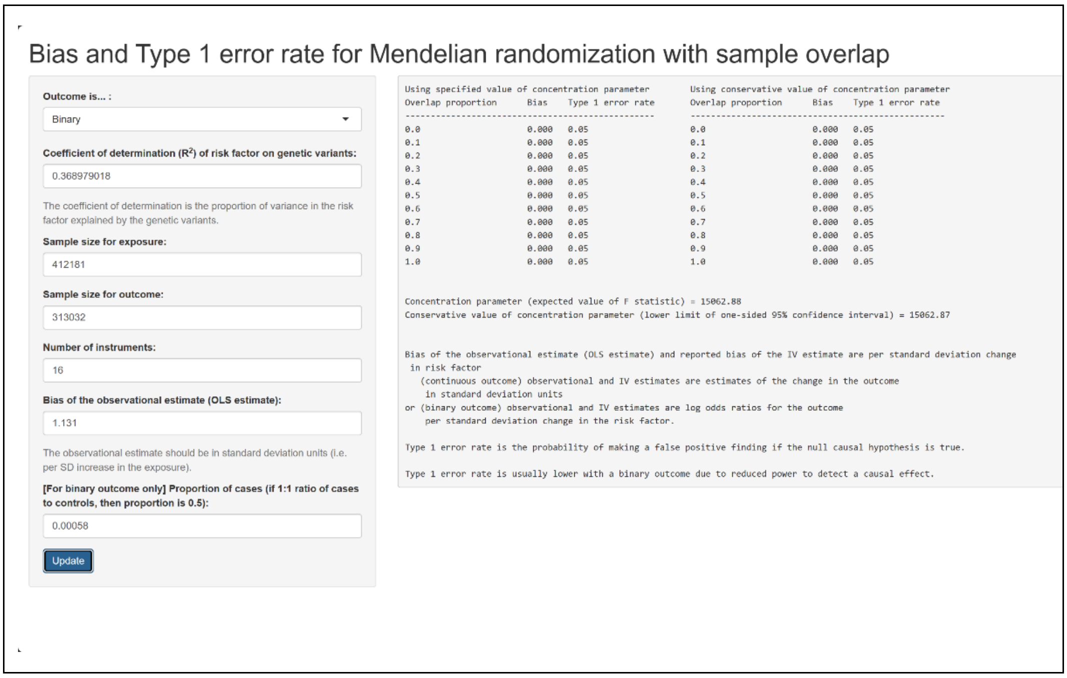

C. Bias and Type 1 error rate for Mendelian randomization with sample overlap of GV-OFP

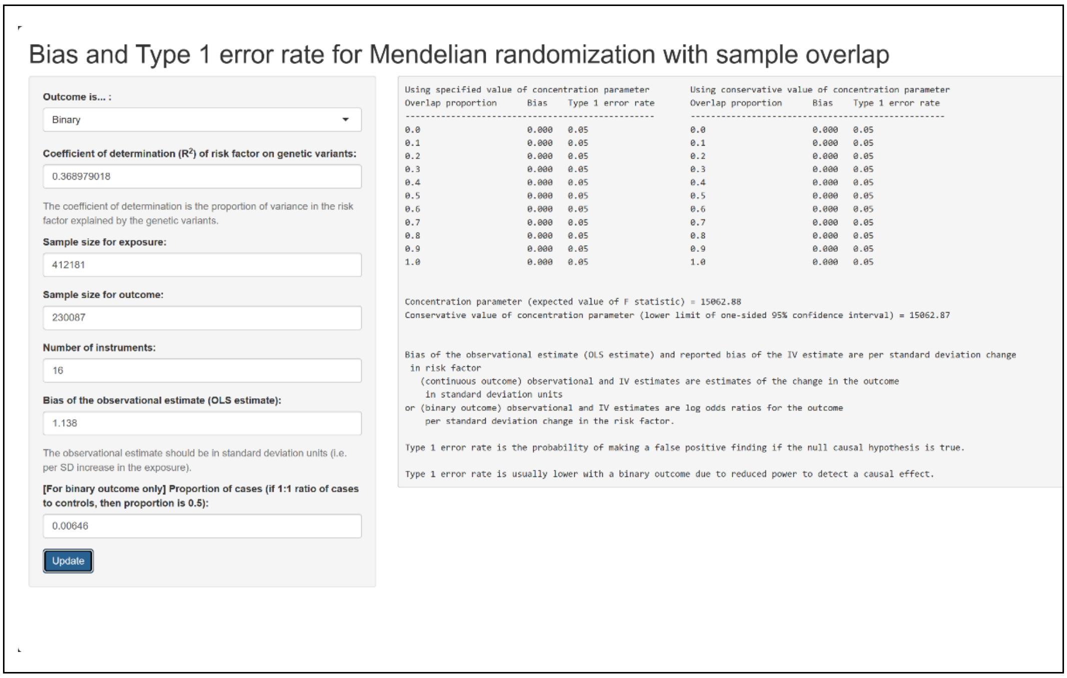

D. Bias and Type 1 error rate for Mendelian randomization with sample overlap of AIHYPER -OP

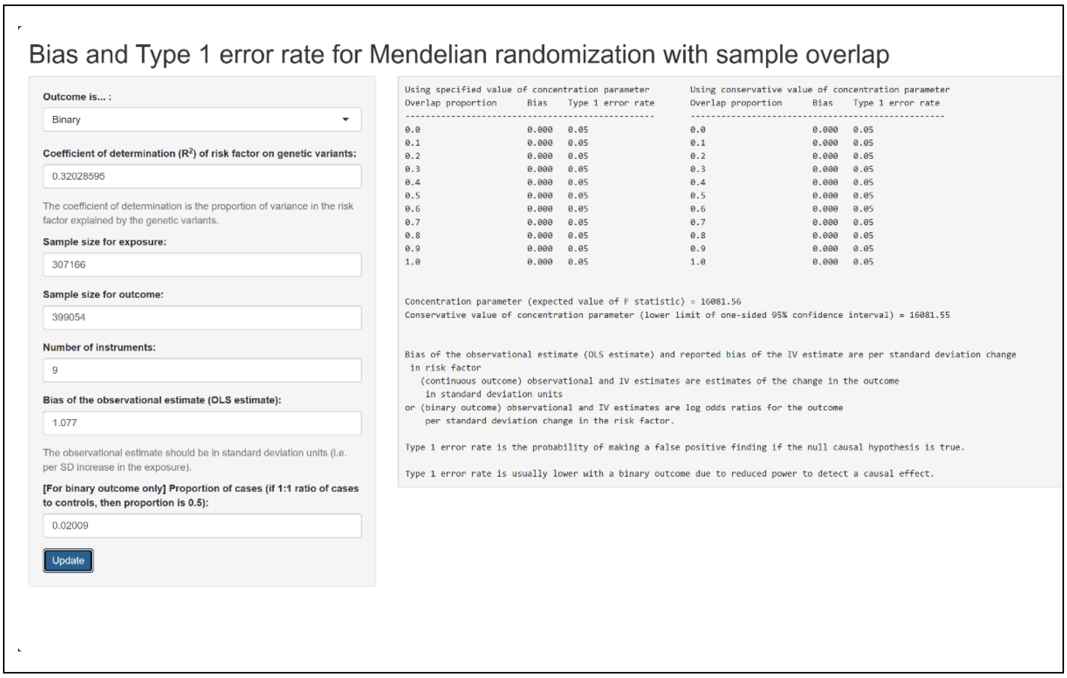

E. Bias and Type 1 error rate for Mendelian randomization with sample overlap of AIHYPER -OF

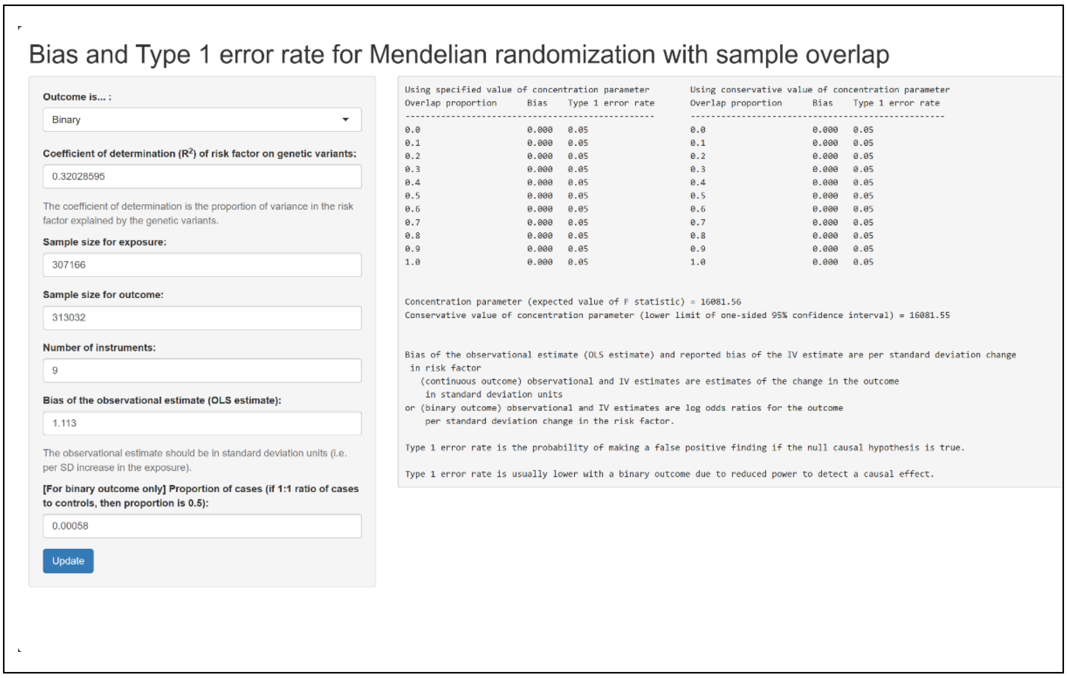

F. Bias and Type 1 error rate for Mendelian randomization with sample overlap of AIHYPER -OFP

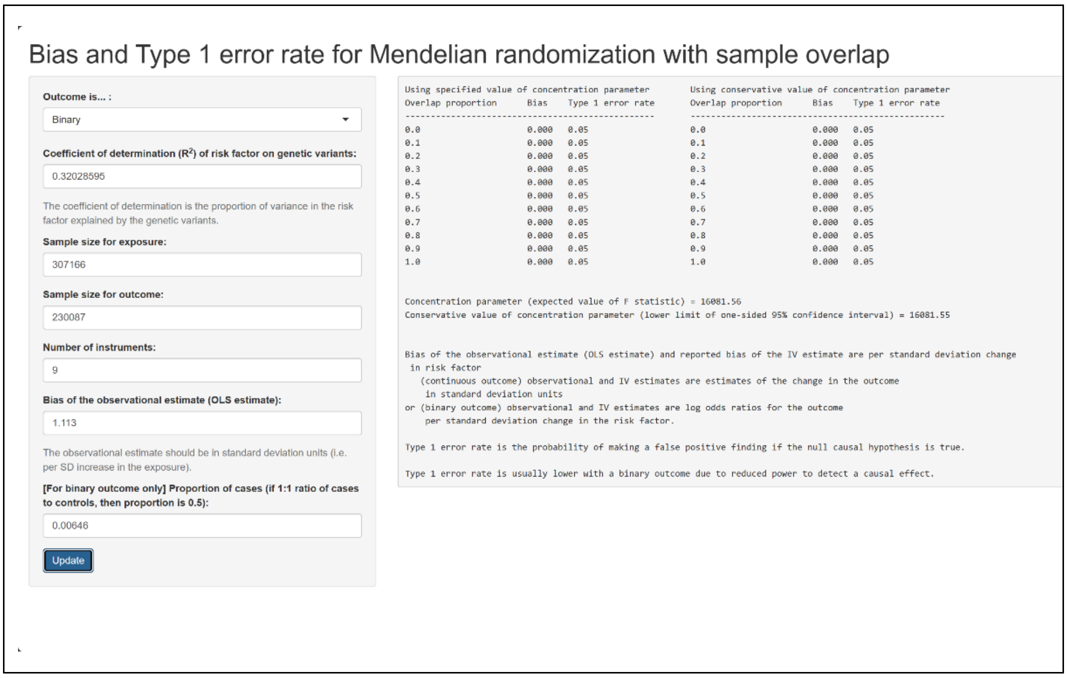

G. Bias and Type 1 error rate for Mendelian randomization with sample overlap of HYPOD -OP

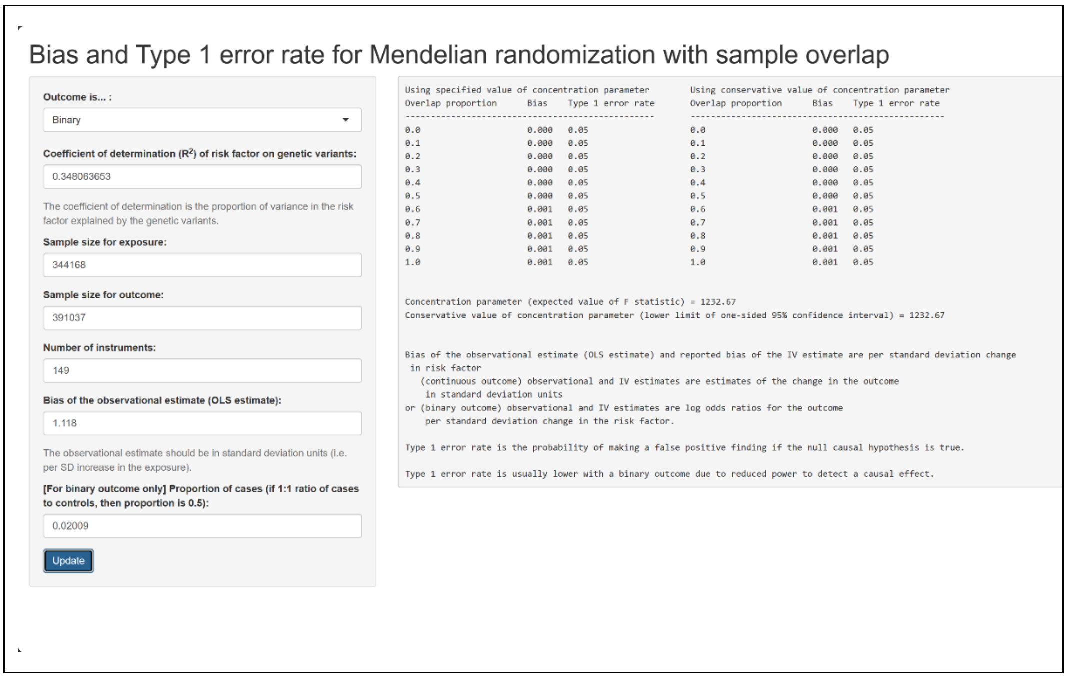

H. Bias and Type 1 error rate for Mendelian randomization with sample overlap of HYPOD -OF

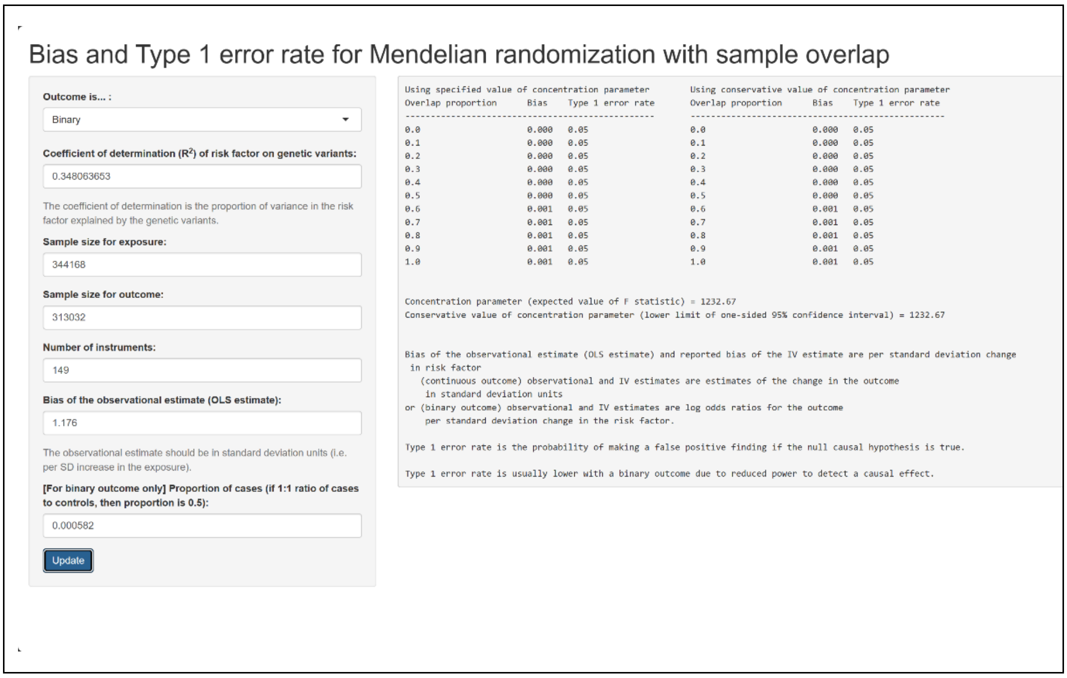

I. Bias and Type 1 error rate for Mendelian randomization with sample overlap of HYPOD -OFP

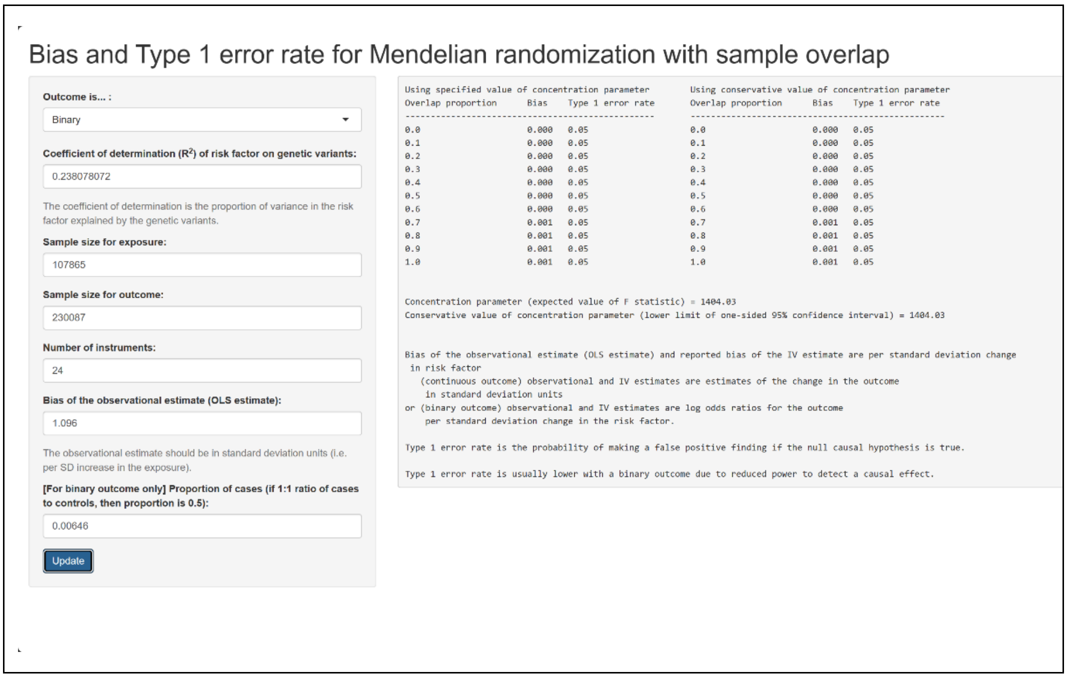

J. Bias and Type 1 error rate for Mendelian randomization with sample overlap of AIHYPO -OP

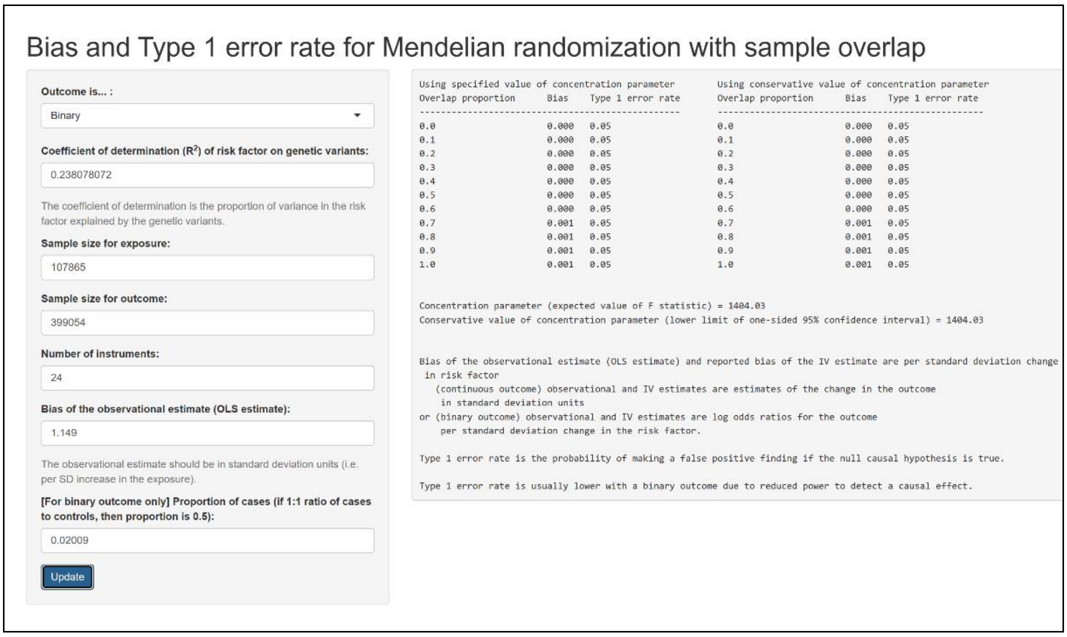

K. Bias and Type 1 error rate for Mendelian randomization with sample overlap of HYPOD -OF

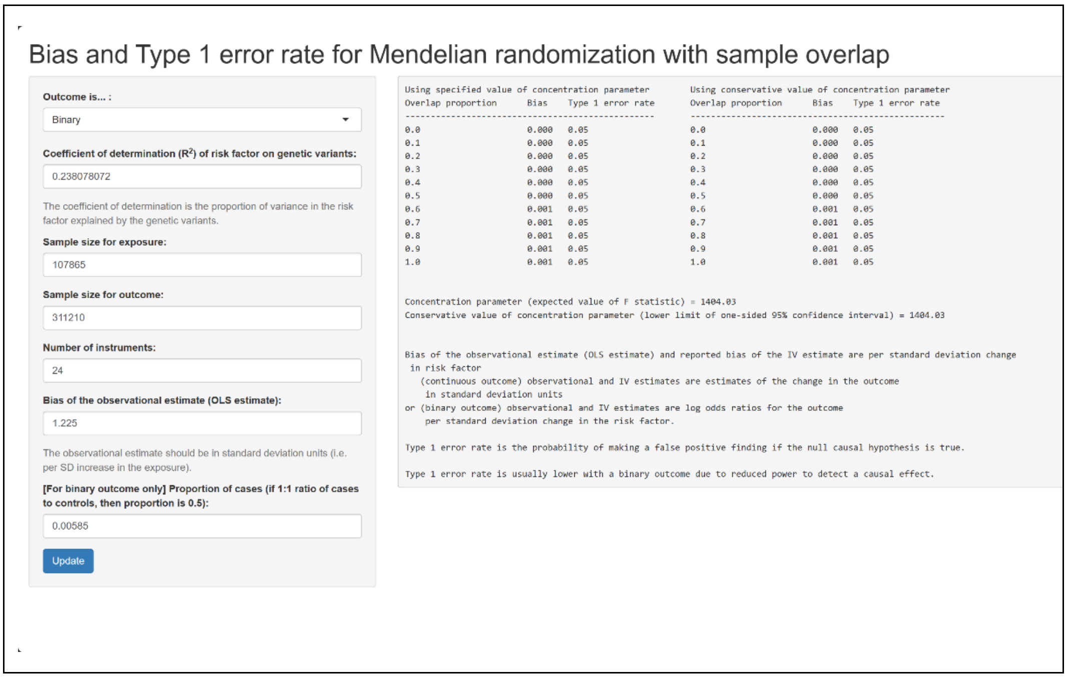

Bias and Type 1 error rate for Mendelian randomization with sample overlap

Outcome is... :

Binary

Coefficient of determination (R^2) of risk factor on genetic variants:

0.238078072

The coefficient of determination is the proportion of variance in the risk factor explained by the genetic variants.

Sample size for exposure:

107865

Sample size for outcome:

311210

Number of instruments:

24

Bias of the observational estimate (OLS estimate):

1.225

The observational estimate should be in standard deviation units (i.e. per SD increase in the exposure).

[For binary outcome only] Proportion of cases (if 1:1 ratio of cases to controls, then proportion is 0.5):

0.00585

Update

Using specified value of concentration parameter			Using conservative value of concentration parameter		
Overlap proportion	Bias	Type 1 error rate	Overlap proportion	Bias	Type 1 error rate
0.0	0.000	0.05	0.0	0.000	0.05
0.1	0.000	0.05	0.1	0.000	0.05
0.2	0.000	0.05	0.2	0.000	0.05
0.3	0.000	0.05	0.3	0.000	0.05
0.4	0.000	0.05	0.4	0.000	0.05
0.5	0.000	0.05	0.5	0.000	0.05
0.6	0.001	0.05	0.6	0.001	0.05
0.7	0.001	0.05	0.7	0.001	0.05
0.8	0.001	0.05	0.8	0.001	0.05
0.9	0.001	0.05	0.9	0.001	0.05
1.0	0.001	0.05	1.0	0.001	0.05

Concentration parameter (expected value of F statistic) = 1404.03
Conservative value of concentration parameter (lower limit of one-sided 95% confidence interval) = 1404.03

Bias of the observational estimate (OLS estimate) and reported bias of the IV estimate are per standard deviation change in risk factor
(continuous outcome) observational and IV estimates are estimates of the change in the outcome in standard deviation units
or (binary outcome) observational and IV estimates are log odds ratios for the outcome per standard deviation change in the risk factor.

Type 1 error rate is the probability of making a false positive finding if the null causal hypothesis is true.

Type 1 error rate is usually lower with a binary outcome due to reduced power to detect a causal effect.

L. Bias and Type 1 error rate for Mendelian randomization with sample overlap of HYPOD -OFP

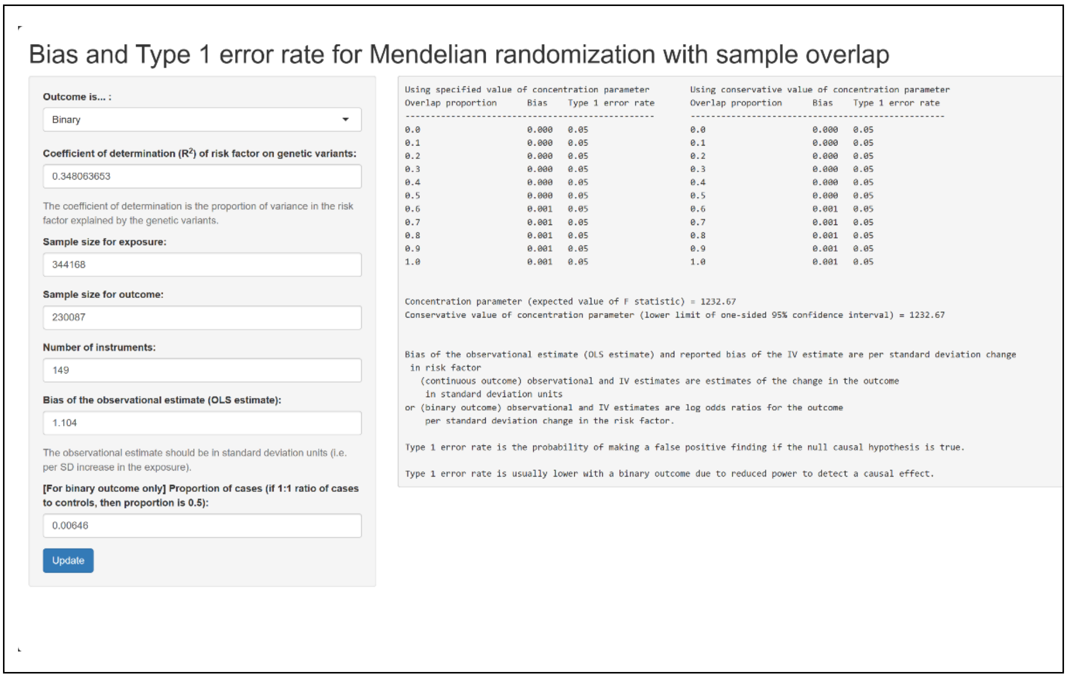

Bias and Type 1 error rate for Mendelian randomization with sample overlap

Outcome is... :

Binary

Coefficient of determination (R^2) of risk factor on genetic variants:

0.348063653

The coefficient of determination is the proportion of variance in the risk factor explained by the genetic variants.

Sample size for exposure:

344168

Sample size for outcome:

230087

Number of instruments:

149

Bias of the observational estimate (OLS estimate):

1.104

The observational estimate should be in standard deviation units (i.e. per SD increase in the exposure).

[For binary outcome only] Proportion of cases (if 1:1 ratio of cases to controls, then proportion is 0.5):

0.00646

Update

Using specified value of concentration parameter			Using conservative value of concentration parameter		
Overlap proportion	Bias	Type 1 error rate	Overlap proportion	Bias	Type 1 error rate
0.0	0.000	0.05	0.0	0.000	0.05
0.1	0.000	0.05	0.1	0.000	0.05
0.2	0.000	0.05	0.2	0.000	0.05
0.3	0.000	0.05	0.3	0.000	0.05
0.4	0.000	0.05	0.4	0.000	0.05
0.5	0.000	0.05	0.5	0.000	0.05
0.6	0.001	0.05	0.6	0.001	0.05
0.7	0.001	0.05	0.7	0.001	0.05
0.8	0.001	0.05	0.8	0.001	0.05
0.9	0.001	0.05	0.9	0.001	0.05
1.0	0.001	0.05	1.0	0.001	0.05

Concentration parameter (expected value of F statistic) = 1232.67
Conservative value of concentration parameter (lower limit of one-sided 95% confidence interval) = 1232.67

Bias of the observational estimate (OLS estimate) and reported bias of the IV estimate are per standard deviation change in risk factor
(continuous outcome) observational and IV estimates are estimates of the change in the outcome in standard deviation units
or (binary outcome) observational and IV estimates are log odds ratios for the outcome per standard deviation change in the risk factor.

Type 1 error rate is the probability of making a false positive finding if the null causal hypothesis is true.

Type 1 error rate is usually lower with a binary outcome due to reduced power to detect a causal effect.

M. Bias and Type 1 error rate for Mendelian randomization with sample overlap of NT -OP

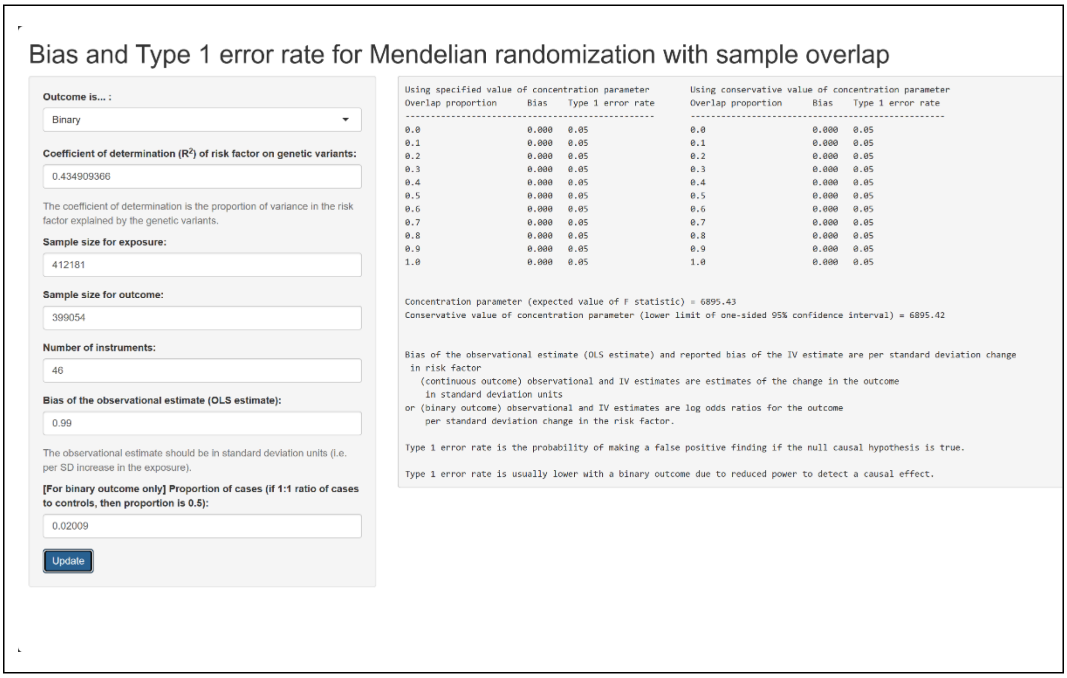

N. Bias and Type 1 error rate for Mendelian randomization with sample overlap of HYPOD -OF

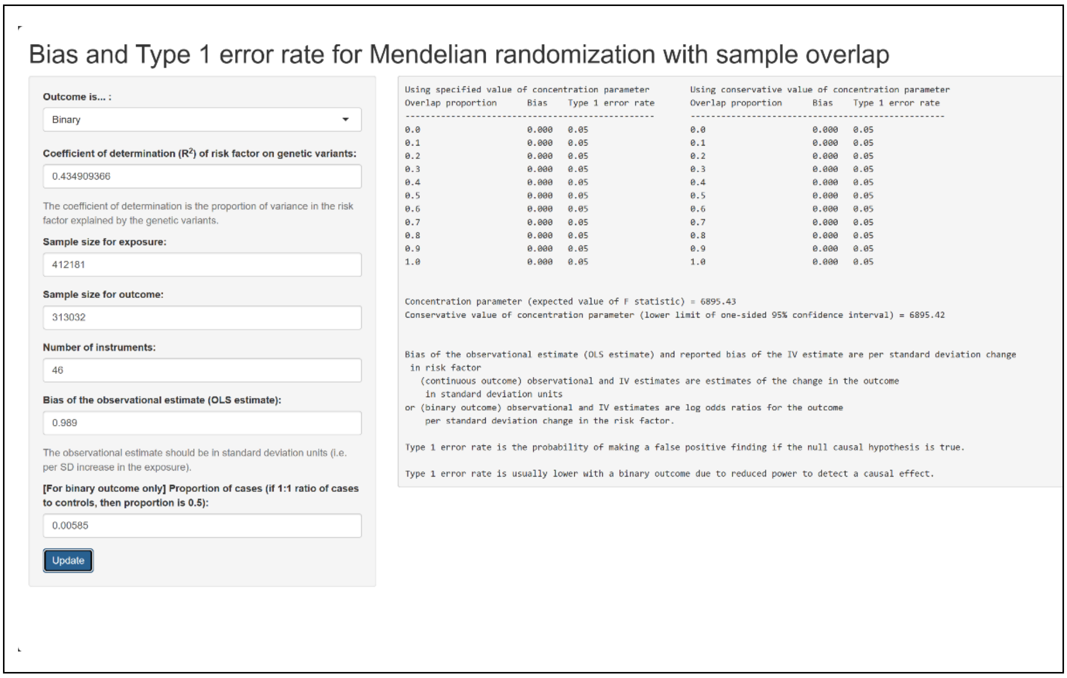

O. Bias and Type 1 error rate for Mendelian randomization with sample overlap of HYPOD -OFP

Bias and Type 1 error rate for Mendelian randomization with sample overlap

Outcome is... :

Binary

Coefficient of determination (R^2) of risk factor on genetic variants:

0.434909366

The coefficient of determination is the proportion of variance in the risk factor explained by the genetic variants.

Sample size for exposure:

230087

Sample size for outcome:

313032

Number of instruments:

46

Bias of the observational estimate (OLS estimate):

0.984

The observational estimate should be in standard deviation units (i.e. per SD increase in the exposure).

[For binary outcome only] Proportion of cases (if 1:1 ratio of cases to controls, then proportion is 0.5):

0.00646

Update

```
Using specified value of concentration parameter       Using conservative value of concentration parameter
Overlap proportion      Bias    Type 1 error rate      Overlap proportion      Bias    Type 1 error rate
------------------------------------------------       ------------------------------------------------
0.0                     0.000   0.05                   0.0                     0.000   0.05
0.1                     0.000   0.05                   0.1                     0.000   0.05
0.2                     0.000   0.05                   0.2                     0.000   0.05
0.3                     0.000   0.05                   0.3                     0.000   0.05
0.4                     0.000   0.05                   0.4                     0.000   0.05
0.5                     0.000   0.05                   0.5                     0.000   0.05
0.6                     0.000   0.05                   0.6                     0.000   0.05
0.7                     0.000   0.05                   0.7                     0.000   0.05
0.8                     0.000   0.05                   0.8                     0.000   0.05
0.9                     0.000   0.05                   0.9                     0.000   0.05
1.0                     0.000   0.05                   1.0                     0.000   0.05


Concentration parameter (expected value of F statistic) = 3848.81
Conservative value of concentration parameter (lower limit of one-sided 95% confidence interval) = 3848.80


Bias of the observational estimate (OLS estimate) and reported bias of the IV estimate are per standard deviation change
 in risk factor
   (continuous outcome) observational and IV estimates are estimates of the change in the outcome
    in standard deviation units
or (binary outcome) observational and IV estimates are log odds ratios for the outcome
    per standard deviation change in the risk factor.

Type 1 error rate is the probability of making a false positive finding if the null causal hypothesis is true.

Type 1 error rate is usually lower with a binary outcome due to reduced power to detect a causal effect.
```
